# Targeted exon skipping with AAV-mediated split adenine base editors

**DOI:** 10.1038/s41421-019-0109-7

**Published:** 2019-08-20

**Authors:** Jackson Winter, Alan Luu, Michael Gapinske, Sony Manandhar, Shraddha Shirguppe, Wendy S. Woods, Jun S. Song, Pablo Perez-Pinera

**Affiliations:** 1 Department of Bioengineering, University of Illinois at Urbana-Champaign, Urbana, IL 61801 USA; 2 Department of Physics, University of Illinois at Urbana-Champaign, Urbana, IL 61801 USA; 3 Carl R. Woese Institute for Genomic Biology, University of Illinois at Urbana-Champaign, Urbana, IL 61801 USA; 4 Carle Illinois College of Medicine, Champaign, IL 61820 USA; 5 Cancer Center at Illinois, University of Illinois at Urbana-Champaign, Urbana, IL 61801 USA

**Keywords:** Biological techniques, Transcription

## Abstract

Techniques for exclusion of exons from mature transcripts have been applied as gene therapies for treating many different diseases. Since exon skipping has been traditionally accomplished using technologies that have a transient effect, it is particularly important to develop new techniques that enable permanent exon skipping. We have recently shown that this can be accomplished using cytidine base editors for permanently disabling the splice acceptor of target exons. We now demonstrate the application of CRISPR-Cas9 adenine deaminase base editors to disrupt the conserved adenine within splice acceptor sites for programmable exon skipping. We also demonstrate that by altering the amino acid sequence of the linker between the adenosine deaminase domain and the Cas9-nickase or by coupling the adenine base editor with a uracil glycosylase inhibitor, the DNA editing efficiency and exon-skipping rates improve significantly. Finally, we developed a split base editor architecture compatible with adeno-associated viral packaging. Collectively, these results represent significant progress toward permanent in vivo exon skipping through base editing and, ultimately, a new modality of gene therapy for the treatment of genetic diseases.

## Introduction

Exon splicing is a natural process that occurs during mRNA maturation and results in exclusion of intronic sequences and the assembly of consecutive or nonconsecutive exons from pre-mRNA^1^. The capability to program transcript splicing is highly desirable for synthetic biology and therapeutic applications, specifically for the treatment of monogenic diseases. Since autosomal diseases are often caused by mutations within exons that lead to loss of protein function, removal of the affected exon may provide a therapeutic benefit by enabling translation of truncated protein isoforms free of mutations that are capable of partially fulfilling the physiological role of the full-length protein. Programmable exon skipping has been demonstrated to be an effective treatment option for diseases such as muscular dystrophies^2^, epidermolysis bullosa^3^, and spinal muscular atrophy^4^.

Conventional targeted exon skipping has been accomplished by directing antisense oligonucleotides (AONs) to splicing regulatory elements in order to block the native splicing machinery and prevent incorporation of the targeted exon into the mature transcript^5^. AONs are typically delivered by local injection and, given their transient nature, necessitate repeated administration to achieve a lasting therapeutic benefit. More recently, the CRISPR–Cas9 genome editing system has been shown to induce permanent exon skipping^6^, which has been harnessed for therapeutic correction of genetic diseases^7^. However, these gene editing approaches rely on introduction of double strand breaks (DSBs) and, while the targeted exons were effectively skipped, repair of DSBs can result in unpredictable phenotypic outcomes^8^, including a DNA damage response involving activation of *TP53* that can compromise survival of the edited cells and limit the therapeutic benefit^9,10^ or even introduce potentially pathogenic translocations^11^.

One recently developed technology that can overcome these problems is single base editing. Single base editors utilize a deaminase domain fused to a Cas9-nickase that can be directed by a single-guide RNA (sgRNA) to introduce targeted C > T^12^ or A > G^13^ conversions within a small, user-defined window. By enabling targeted mutagenesis without nuclease activity, base editors offer a promising platform for minimally disruptive and permanent exon-skipping therapies. We and others have previously demonstrated the application of C > T base editors to induce programmable exon skipping through mutation of the conserved guanine residue preceding each exon, by targeting the cytosine on the opposite strand^14,15^. We estimated that the number of inner exons that can be targeted by this approach, termed CRISPR-SKIP, is 118,089 out of 187,636 inner exons. However, given the highly conserved sequence of splice acceptors, some CRISPR-SKIP target sites have low predicted on-target or high off-target scores^14^. In this work, we sought to increase the number of exons that can be targeted with high efficiency and specificity by adding a novel editing tool to the CRISPR-SKIP toolbox. More specifically, the experiments in this paper describe the application of A > G base editors (ABE) to induce exon skipping by mutating the adenine in the highly conserved AG dinucleotide within splice acceptors. Since therapeutic applications of exon skipping require achieving modification rates that surpass certain thresholds, we optimized the ABE performance by modifying the linker tethering the deaminase domain and the Cas9 scaffold. Finally, to enable in vivo applications of CRISPR-SKIP, we developed a split base editor architecture that is compatible with adeno-associated virus (AAV)-mediated delivery.

## Results

**Fig. 1:**
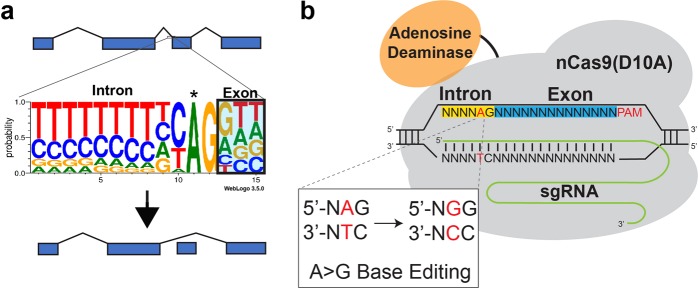
Mutation of the conserved adenine residue of a splice acceptor using an ABE results in exon skipping. **a** Diagram of the consensus sequence of splice acceptor sites. Mutation of the conserved adenosine residue (*) prevents recognition by the splice machinery which leads to skipping of the exon. **b** ABEs can be directed to the splice acceptor site to convert the target A to a G, which results in exon skipping

Nearly all splice acceptors consist of a highly conserved adenosine–guanosine dinucleotide at the 5′ end of the exon (Fig. 1a)^16^. We hypothesized that conversion of the adenosine to a guanosine by targeting with ABEs^13^ would prevent recognition of the exon by the spliceosome, thereby triggering its exclusion from mature transcripts (Fig. 1b).

**Fig. 2:**
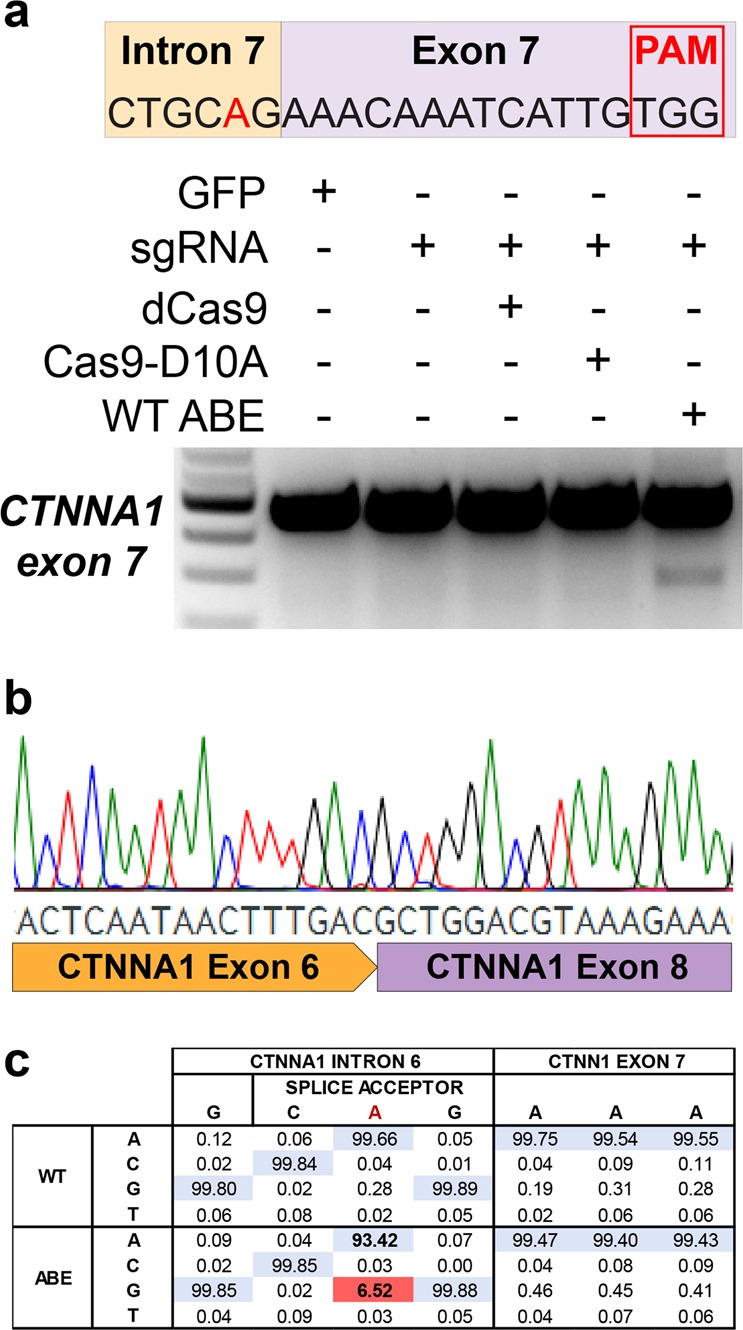
Targeted exon skipping of CTNNA1 Exon 7 using ABE. **a** HEK293T cells were transfected with ABE 7.10 and a sgRNA targeting the splice acceptor site of *CTNNA1* exon 7. Targeted exon skipping was observed after performing RT-PCR that could not be induced by the sgRNA alone, or in combination with dead Cas9 or D10A nickase Cas9. **b** Sanger sequencing of the shorter transcript confirmed exclusion of exon 7 from the *CTNNA1* mature transcript. **c** High-throughput sequencing confirmed targeted A > G mutations in genomic DNA at the *CTNNA1* exon 7 splice acceptor site

To test this hypothesis we targeted an ABE to the splice acceptor site of *CTNNA1* exon 7. Plasmids encoding ABE 7.10, which consists of two engineered *Escherichia coli* TadA adenine deaminase domains fused to a Cas9-D10A nickase^13^ and a sgRNA targeting the splice acceptor, were transfected into HEK293T cells. After 6 days, the RNA was isolated and retrotranscribed to cDNA, which was used in PCRs to detect skipping of exon 7. In samples treated simultaneously with the ABE and the sgRNA, two PCR amplicons were observed, corresponding to the expected size of the full-length mature mRNA and mRNA lacking exon 7. The transcript lacking exon 7 was not observed in samples treated with the sgRNA alone or the sgRNA in combination with dead Cas9 or Cas9-D10A (Fig. 2a). Sanger sequencing of the shorter PCR product confirmed that *CTNNA1* exon 6 was followed immediately by exon 8, confirming that exon 7 was skipped (Fig. 2b). High-throughput sequencing (HTS) of genomic DNA samples transfected with ABE and the sgRNA confirmed successful A > G mutation of the *CTNNA1* exon 7 splice acceptor site in 6.52% of the strands (Fig. 2c).

To determine the optimal time frame needed to achieve maximal rates of exon skipping, we performed a time-course experiment by transfecting HEK293T cells with plasmids encoding ABE 7.10 and the *CTNNA1* exon 7 sgRNA and isolating RNA for analysis at various time points over a 10-day period. The truncated product was readily detectable at day 2 and the rate of exon skipping continued to steadily increase until reaching a plateau at day 6 (Supplementary Fig. S1), which is similar to the optimal time frame for exon skipping with C > T base editors^14^. For all subsequent experiments, samples were analyzed 6 days post transfection. In addition, the relative ratios of plasmids encoding the base editor and the sgRNA were varied to determine the optimal transfection conditions. We found that using 500 ng of sgRNA plasmid in combination with 500 ng of base editor plasmid or 250 ng of base editor plasmid in combination with 750 ng of sgRNA plasmid resulted in the highest rates of exon skipping in HEK293T cells transfected in 24-well plates (Supplementary Fig. S2).

To determine whether the exon skipping induced by ABE is cell line specific, we transfected HEPG2 and HCT116 cell lines with ABE, the *CTNNA1* exon 7 sgRNA or an sgRNA targeting *AHCY* exon 9, respectively. In addition, to determine if the technique worked in other species, we transfected mouse Neuro2A and Hepa1–6 cells with ABE and a sgRNA targeting *CTNNB1* exon 11. The target exon was skipped in all cell lines and only in the ABE-treated samples (Supplementary Fig. S3).

**Fig. 3:**
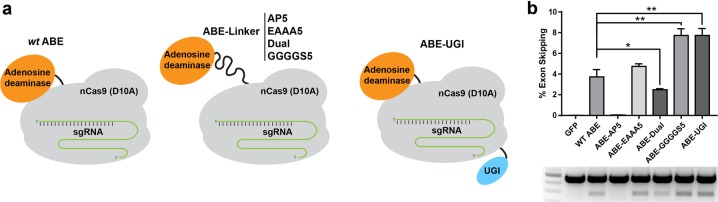
Improvement of ABE editing activity by optimization of the linker between the deaminase and Cas9 or addition of a uracil glycosylase inhibitor domain. **a** Schematic representation of several of the ABE variants that were constructed by either modifying the linker tethering nCas9 and the deaminase domain or by fusing ABE 7.10 with a UGI. **b** High-throughput sequencing of cDNA demonstrated significantly increased levels of exon skipping by several of the ABE variants as compared to ABE 7.10. * and ** correspond to *P* < 0.05 and *P* < 0.01, respectively by two-tailed unpaired Student’s *t* test, *n* = 3

Since AON-induced exon skipping has already been successfully applied for correction of monogenic diseases such as Leber Congenital Amaurosis^17^, atherosclerosis^18^, FTDP-17^19^, cancer^20^, rheumatoid arthritis^21^, Huntington’s disease^5^, dystrophic epidermis bullosa^22^, and Duchenne muscular dystrophy (DMD)^23^, we anticipate that CRISPR-SKIP will have multiple applications in biomedicine. However, correction of monogenic diseases requires production of a critical amount of functional protein product to achieve therapeutic benefit. Even though as little as 4% recovery of dystrophin expression restores significant muscle function to treat DMD^24,25^, a higher modification rate is needed in other cases, such as Huntington disease, which requires a 40% reduction of mutant Huntingtin for clinical improvement. For this reason, we sought to improve the exon-skipping rate induced by ABE by optimizing the amino-acid sequence of the linker between the TadA deaminase domains and the Cas9-D10A. Linkers between different domains in chimeric proteins influence parameters that are critical for protein function such as maintaining protein stability and folding^26^. To explore the effect of the linker domains on editing efficiency, we created ABE constructs with linkers of either five repeats of alanine followed by proline (ABE-AP_5_), five repeats of four glycine residues and a serine (ABE-GGGGS_5_), the linker in ABE 7.10^13^ fused to GGGGS (ABE-Dual), or 5 repeats of glutamic acid followed by three alanine residues (ABE-EAAA_5_) (Fig. 3a). Furthermore, since hypoxanthine is a spontaneous deamination product of adenine and a recently identified family of uracil–DNA glycosylases has been shown to act on hypoxanthine as part of the DNA repair process^27^, we reasoned that fusing ABE with a 83-amino acid uracil glycosylase inhibitor (UGI) domain may enhance DNA editing rate. For this reason, another construct was generated by adding a UGI domain to the C-terminus of the ABE 7.10 (ABE-UGI) (Fig. 3a).

These constructs were transfected separately into HEK293T cells along with the *CTNNA1* exon 7 sgRNA, and rates of exon skipping were measured using RNA-seq (Fig. 3b). Our results demonstrated that ABE with a GGGGS_5_ linker (7.73%, *P* = 0.002) and the EAAA_5_ linker (4.73%, *P* = 0.082) induced exon skipping more efficiently than ABE 7.10 (3.73%). Furthermore, ABE-UGI also outperformed the ABE 7.10 with a skipping efficiency of 7.73% (*P* = 0.002).

**Fig. 4:**
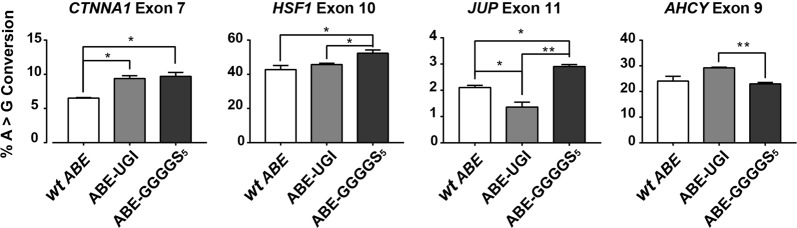
Quantification of genomic DNA mutation rates created by several ABE constructs at multiple target sites. High-throughput sequencing was used to quantify rates of A > G genomic DNA mutation and rates of exon skipping across multiple targets using several ABE variants. * and ** correspond to *P* < 0.05 and *P* < 0.01, respectively by two-tailed unpaired Student’s *t* test across two biological replicates

**Fig. 5:**
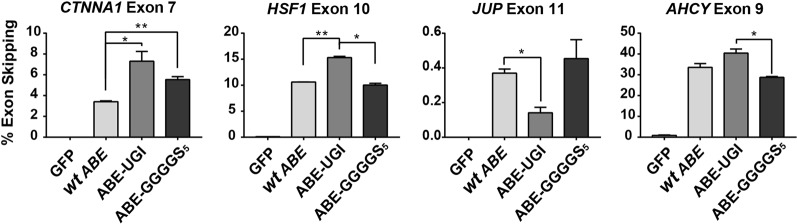
Quantification of exon skipping rates at multiple gene targets induced by several ABE constructs. High-throughput sequencing of cDNA was used to quantify rates of exon skipping across multiple targets using several ABE variants. * and ** correspond to *P* < 0.05 and *P* < 0.01, respectively by two-tailed unpaired Student’s *t* test across two biological replicates

In order to compare the editing efficiencies of these improved ABE variants across multiple targets, as well as to correlate rates of modification in genomic DNA with rates of exclusion of the targeted exon from mRNA transcripts, we performed HTS on both genomic DNA (Fig. 4) and cDNA (Fig. 5) at multiple target sites in cells transfected with ABE 7.10, ABE-GGGGS_5_, and ABE-UGI (Supplementary Table S1). These results confirmed that use of the ABE-GGGGS_5_ and ABE-UGI led to significant increases in both A > G base editing rates and exon-skipping rates over ABE 7.10 for many of the targets that were tested. In these experiments, the highest observed A > G mutation rates for each target were 9.70% by ABE-GGGGS_5_ at *CTNNA1* exon 7, 52.33% by ABE-GGGGS_5_ at *HSF1* exon 11, 2.90% by ABE-GGGGS_5_ at *JUP* exon 10, and 29.23% by ABE-UGI at *AHCY* exon 9 (Fig. 4). The highest observed exon-skipping rates as determined by RNA-seq for each target were 7.30% by ABE-UGI at *CTNNA1* exon 7, 15.31% by ABE-UGI at *HSF1* exon 11, 0.45% by ABE-GGGGS_5_ at *JUP* exon 10 and 40.45% by ABE-UGI at *AHCY* exon 9 (Fig. 5).

**Fig. 6:**
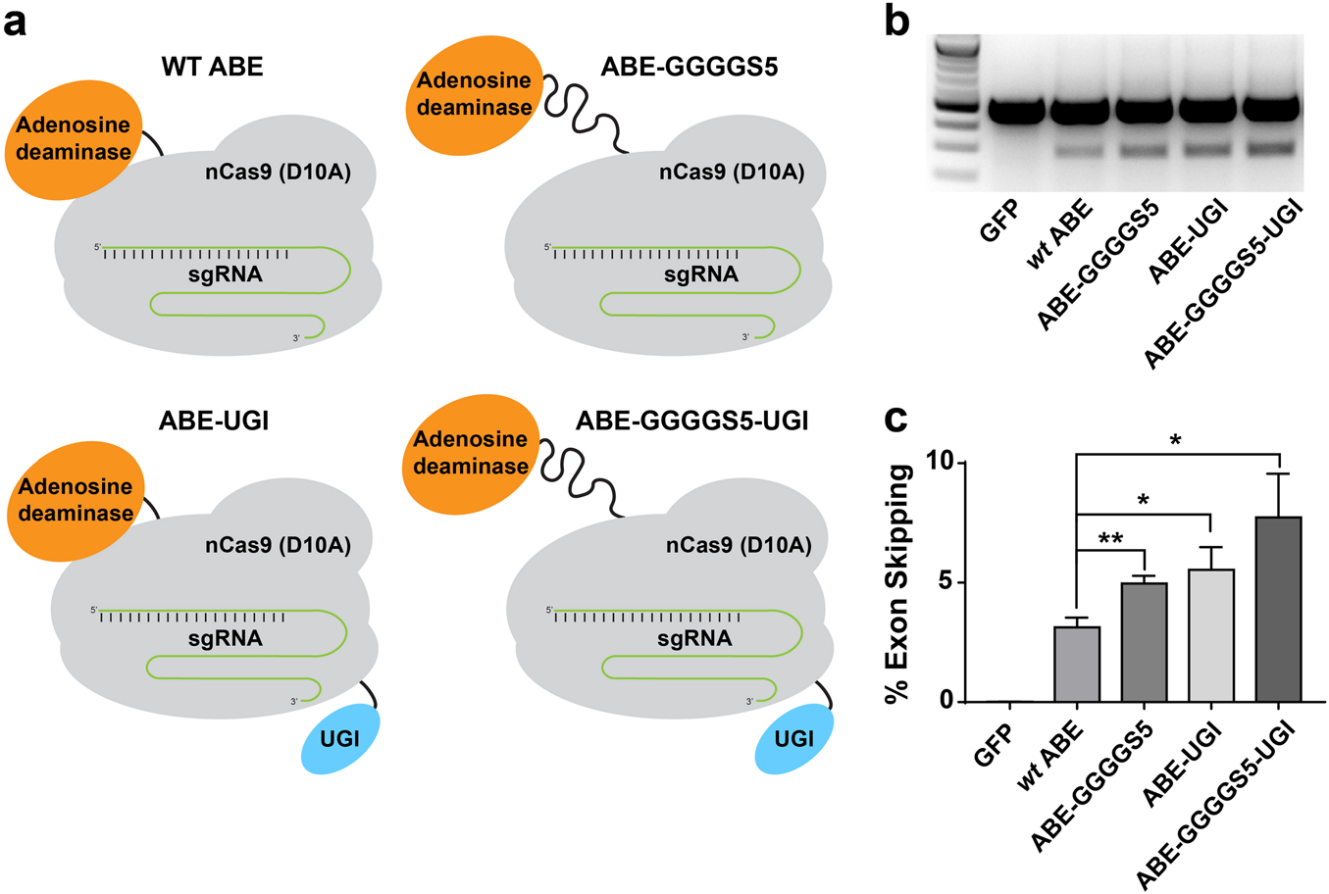
Addition of both a uracil glycosylase inhibitor domain and an optimized linker further increased rates of exon skipping. **a** Schematic representation of the ABE variants constructed by either modifying the linker tethering nCas9 and the deaminase domain, by fusing ABE 7.10 with a UGI or both. **b** Combining the GGGGS_5_ linker and UGI domain within the same ABE construct led to higher rates of exon skipping than the ABEs containing each modification individually, suggesting an increased A > G mutation rates in genomic DNA when both domains are used. **c** High-throughput sequencing analysis of RT-PCR products demonstrated significantly increased levels of exon skipping by several of the ABE variants compared with ABE 7.10. (* and ** correspond to *P* < 0.05 and *P* < 0.01 respectively by two-tailed unpaired Student’s *t* test, *n* = 3)

In an effort to further increase the editing efficiency of the ABE, we created an additional ABE construct containing both the GGGGS_5_ linker and the UGI domain (ABE-GGGGS_5_-UGI) (Fig. 6a). We hypothesized that a base editor with both modifications would be more effective than either ABE-GGGGS_5_ or ABE-UGI. Plasmids encoding each ABE were transfected separately into HEK293T cells along with the *CTNNA1* exon 7 sgRNA. Rates of exon skipping were measured by reverse transcription polymerase chain reaction (RT-PCR) (Fig. 6b) and compared using HTS (Fig. 6c). In this set of experiments, ABE-GGGGS_5_-UGI induced a higher rate of exon skipping than all other constructs tested with 7.73% compared to 3.13% for ABE 7.10 (*P* = 0.013), 4.96% for ABE-GGGGS_5_ (*P* = 0.061), and 5.53% for ABE-UGI (*P* = 0.139).

We next sought to determine if the length of the linker between the deaminase domain and Cas9-D10A had any effect on the base editing window within the protospacer. We created ABE constructs with 1–7 repeats of the amino-acid sequence GGGGS. These constructs were then transfected into HEK293T cells along with one of two A-rich sgRNAs targeting the GAPDH locus (Supplementary Table S4). After 3 days, genomic DNA was harvested and the editing rates of each of the As within the protospacer were evaluated for each construct (Supplementary Fig. S4). Interestingly, the editing window expanded towards the 5′ direction of the protospacer for each of the GGGGS constructs compared to ABE 7.10 and resulted in editing of the adenine in position 4, which was not observed with ABE 7.10. Furthermore, the editing efficiencies for positions 4 and 5 increased as the linker length decreased, with ABE-GGGGS_1_ yielding the highest rates of base editing for these positions.

While improving editing efficiency addresses some of the limitations for therapeutic applications using single-base editors, the large size of the DNA constructs encoding ABE remains a significant roadblock to in vivo therapies that rely on gene delivery using AAVs. AAV offers a promising and safe delivery vehicle for gene therapy due to their ability to infect a broad range of cells, including nondividing cells, without eliciting more than a mild-immune response^28^. In addition, they do not integrate into the host genome, thus reducing the risk of disrupting native gene function^29^. However, a major drawback of using AAVs is that the size of the transgene is limited to 4.7 kb for efficient expression^30^, which prevents the packaging of an ABE.

One strategy that can be used to overcome these limitations is splitting the ABE transgene into two separate vectors through the use of *Rhodothermus marinus* inteins, which when expressed as proteins are able to dimerize and cleave themselves out, leaving a near seamless fusion of the two gene products^31^. Here, we tested whether ABEs split in two separate expression cassettes using inteins are active in cultured cells. First, the ABE 7.10 open reading frame was split at the aspartic acid residue at amino-acid position 1109 into two plasmids. The N-terminal plasmid contained the TadA domains, the ABE 7.10 linker, and the first 712 amino acids of Cas9-D10A, followed immediately by an N-terminal intein sequence (N-ABE). The second construct contained a C-terminal intein sequence followed by the remaining 666 amino acids of ABE 7.10 (C-ABE) (Supplementary Fig. S5 and Supplemental Sequences). After transfecting HEK293T cells with the *HSF1* exon 11 sgRNA with the N-ABE plasmid and C-ABE plasmid, we observed exon skipping only in the samples containing both N- and C-terminus split base editor plasmids or the full-length ABE plasmid, which was transfected as control. We did not observe exon skipping levels above background in cells transfected with just the N-terminus or the C-terminus split ABE (Supplementary Fig. S6). Surprisingly, RNA-seq revealed that the rate of exon skipping induced by split ABE (31.98%) was higher that the skipping rate measured in samples transfected with ABE 7.10 (26.23%) (*P* = 0.0019), despite a potentially unfavorable reaction kinetic (Supplementary Fig. S7).

**Fig. 7:**
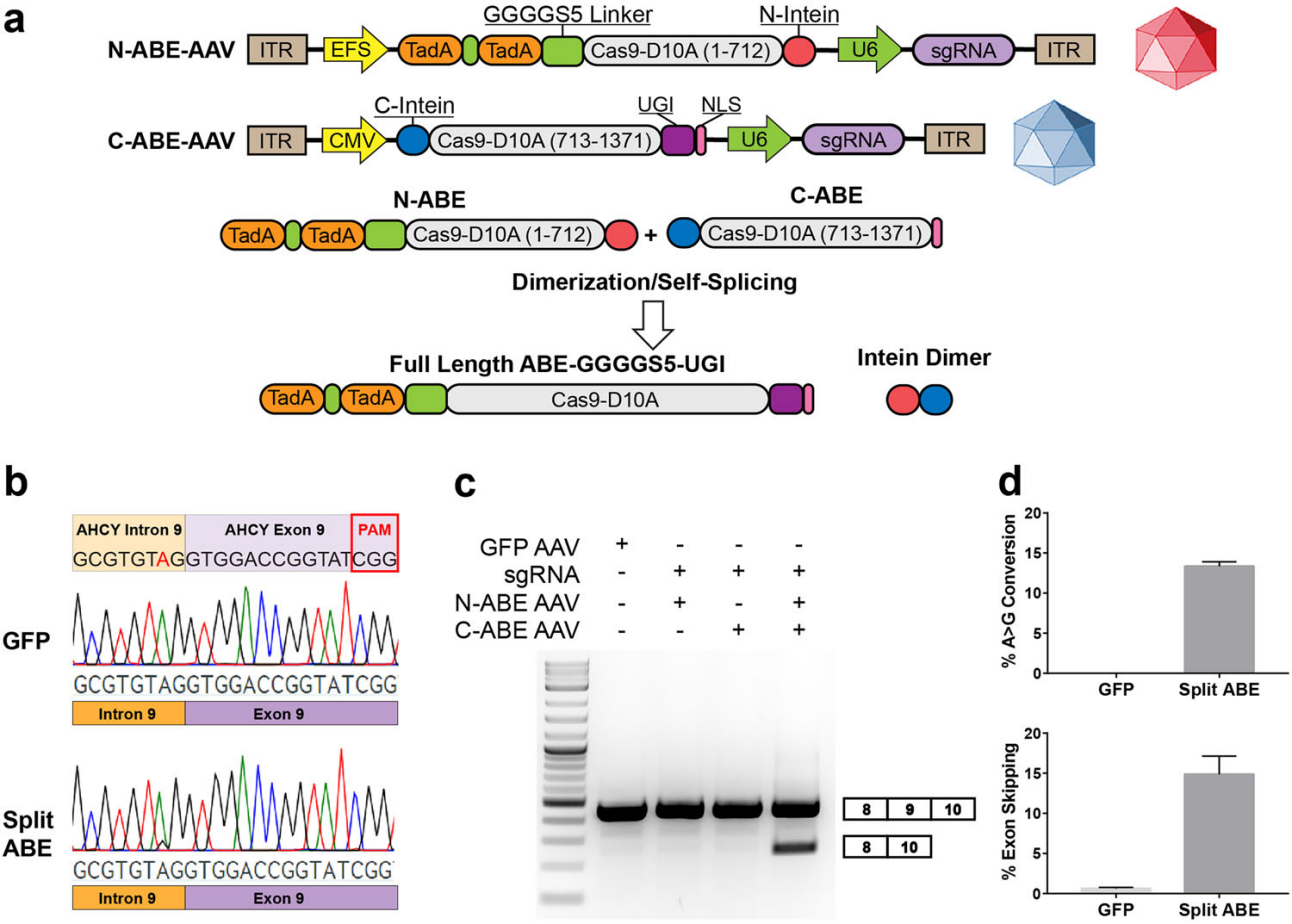
Intein-mediated split ABE facilitates targeted exon skipping following delivery with AAV. **a** Schematics of the split-ABE AAV system. N-terminal and C-terminal intein sequences reconstitute the full-length protein when co-expressed within the cell. **b** Sanger sequencing traces from genomic DNA prepared from HEK293T cells transduced with either GFP-AAV or both N-ABE AAV and C-ABE AAV. A > G mutations are only observed when both N-ABE AAV and C-ABE AAV particles are delivered. **c** RT-PCR products confirm that exon skipping only occurs when both N-ABE AAV and C-ABE AAV are co-delivered. **d** Quantification of A > G mutation rates in the samples described in 7b using EditR (*n* = 3) and **e** exon-skipping rates by densitometry analysis of RT-PCR products (*n* = 3)

We then tested whether these constructs can be packaged into separate AAV particles and co-delivered to achieve base editing and subsequent exon skipping. We split the open reading frame of ABE-GGGGS_5_-UGI at the same residue in Cas9-D10A as before, and cloned the separate constructs between AAV inverted terminal repeats (ITRs) (Fig. 7a). An sgRNA expression cassette under the control of a U6 promoter was also cloned between the ITRs of each construct to enable simultaneous delivery of the sgRNA. After cloning a sgRNA targeting AHCY exon 9 into these plasmids, they were packaged into AAV and used to transduce HEK293T cells. Cells were transduced with the N-ABE AAV, the C-ABE AAV, or both. After 6 days we harvested the cells and confirmed A > G mutations in genomic DNA and exon skipping only in the samples that were treated with both the N-ABE AAV and the C-ABE AAV (Fig. 7b, c). Analysis of genomic DNA from three independent experiments revealed A > G modification rates of 13.33% (Fig. 7d), while densitometry analysis of RT-PCR products of the same samples revealed exon skipping rates of 14.85% (Fig. 7e).

**Fig. 8:**
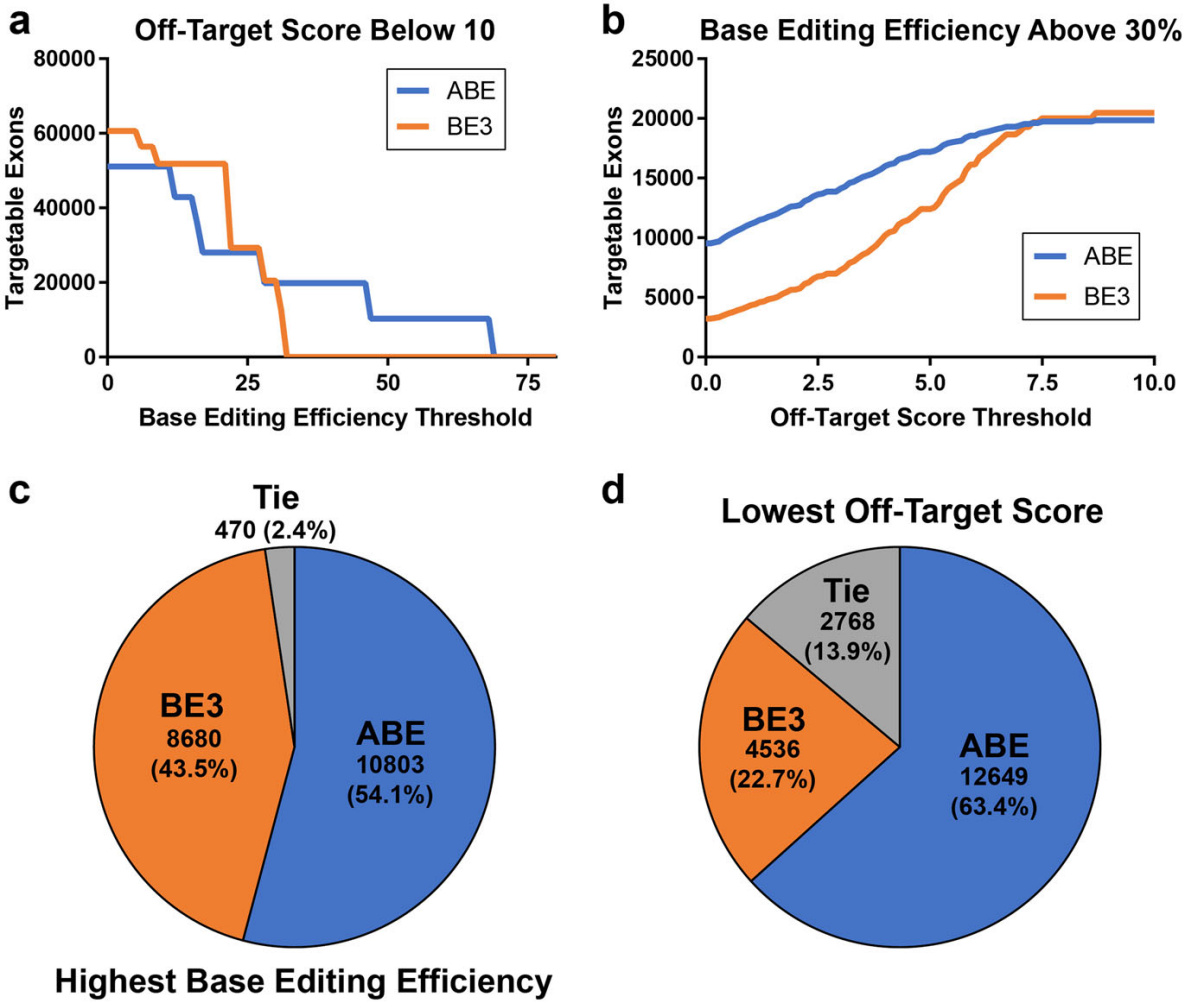
ABE is predicted to have improved on-target and off-target editing efficiencies compared to BE3 when targeting splice acceptor sites. **a** Genome-wide computational analysis of the number of inner exons that can be targeted by ABE and BE3 with predicted editing efficiency of the target base at or above the value on the *x*-axis. Only sgRNAs with an off-target score below ten were considered. **b** Estimation of the number of inner exons that can be targeted by ABE and BE3 using sgRNAs with off-target scores at or below the value on the *x*-axis. Only sgRNAs with an on-target base editing efficiency above 30% were considered. **c** A total of 19,953 inner exons in the human genome can be targeted by both ABE and BE3. The sgRNAs needed to induce skipping with ABE have higher predicted base editing efficiency for targeting 54.1% of the exons, **d** and lower predicted off-target score for targeting 63.4% of the exons

To determine the contribution of ABE editors to the CRISPR-SKIP toolbox, we measured the number of inner exons that could be targeted by ABE using genome-wide computational analysis of PAMs compatible with exon skipping through mutation of the adenosine in the splice acceptor. In this analysis, when only highly specific sgRNAs with off-target scores^32^ at or below 10 were considered, we determined that the number of exons targetable by ABE is higher than the number of exons targeted by BE3 for all base editing efficiency thresholds over 30 (Fig. 8a). Furthermore, the numbers of exons that can be targeted by ABE with an off-target threshold lower than 7.5 is larger than the number that can be targeted with BE3 for on-target base editing efficiency above 30% (Fig. 8b). There are 19,953 inner exons in the human genome that can be targeted by both ABE and BE3. ABE provides higher predicted efficiency for targeting 10,803 of these exons (54.1%) (Fig. 8c) and higher specificity in 12,649 inner exons (63.4%) (Fig. 8d). These results support that ABE not only expands the number of exons that can be targeted by CRISPR-SKIP, but also enables increasing the efficiency and specificity of CRISPR-SKIP.

Collectively, the work described in this paper establishes a platform for inducing exon skipping by mutation of the conserved adenosine in splice acceptors. Since this modification is introduced in genomic DNA, the resulting alternative splicing is expected to be permanent, which provides an important therapeutic advantage over exon-skipping oligonucleotides. However, one of the concerns with gene editing technologies, including base editing, has been the possibility of unintended off-target mutations in regions of the genome that are similar to the target sequence^33^. To investigate the incidence of off-target mutations we analyzed genomic DNA at four predicted off-target locations^32^ for each sgRNA tested by HTS to detect possible mutations (Supplementary Table S2). We only observed off-target A > G mutations at one site within a noncoding region, which was introduced by the *JUP* exon 10 sgRNA. Notably, this sgRNA had the highest predicted off-target score of all sgRNA tested in this work and the mutation rate was low (∼0.5%).

## Discussion

The work described in this paper significantly improves the CRISPR-SKIP toolbox not only by increasing the number of potential exons that can be targeted, but also by engineering a base editing system that is compatible with in vivo delivery by AAV.

Mutation of the adenosine in the conserved splice acceptor AG dinucleotide preceding the exon was predicted to prevent recognition of the targeted exon by the spliceosome machinery; therefore, we anticipated a linear relationship between splice acceptor site mutation rate and exon-skipping rate across targets. However, this was not the case. For example, at *AHCY* exon 9 mutation rates of ∼20% in genomic DNA resulted in skipping rates of ∼50%, whereas at *HSF1* exon 11, mutation rates of ∼50% in genomic DNA resulted in rates of exon skipping of only ∼20%. Since one of the major blocks during transcript elongation is the splicing junction^34–36^, which leads to transient polymerase pausing at the splice sites^37^, it is reasonable to expect that the rate of exon skipping can be higher than the conversion rate measured in genomic DNA. While this is what we observed at most targets, the opposite was observed when we targeted *HSF1*. One potential explanation is that *HSF1* is expressed at low levels in HEK293T cells and it is possible that a time frame longer than 6 days might be needed for the changes in genomic DNA to be reflected in the transcriptome. While it is difficult to predict the exact reason for the overall lack of correlation, we noted that when we studied each target site individually, there was indeed a correlation between the rate of mutation in genomic DNA and exon skipping in mRNA, with higher rates of splice acceptor mutations corresponding to higher rates of exon skipping for all targets except for *HSF1* (Supplementary Fig. S8).

Overall, the observed genomic DNA modification rates and exon skipping rates appeared to vary widely among targets. By analyzing the on-target activity of each guide using *wt* active Cas9, we observed that each of the guides was able to create indels at rates ranging from ~11% to ~30% at the target site demonstrating that each sgRNA was active (Supplementary Table S3). However, *wt* Cas9 nuclease activity did not correlate with ABE editing rates when using the same sgRNA, thus suggesting that the adenine deaminase domain imposes other editing restrictions that are not well understood yet while underscoring the need to perform further studies to develop computational tools for more effectively predicting optimal ABE target sites. While the results of our in silico screening in Fig. 8 show that A > G base editors often have greater predicted on-target editing efficiency and better predicted off-target editing profiles than BE3, it is important to evaluate both base editors at the target site experimentally, as actual results will likely be dependent on local sequence and chromatin context.

Interestingly, the linker optimization studies that we performed identified ABE variants that were more active than the ABE 7.10. However, the improvement was target specific, and we did not identify one single linker variant that was more effective across all targets. It is worth noting that the shorter GGGGS linker variants are able to achieve higher rates of exon skipping for bases located toward the 5′ of the protospacer compared to the longer GGGGS linker variants or ABE 7.10. These results suggest that the best variant to use is dependent on the position of the target A within the protospacer and the sequence context, and could explain why the greatest increases in base editing and exon skipping were seen for *CTNNA1* exon 7 and *JUP* exon 11, as the splice acceptor adenine was located at position 5 of the protospacer, while the target adenine for *HSF1* exon 10 and *AHCY* exon 9 was located at positions 6 and 7, respectively.

Importantly, we also demonstrated that coupling UGI at the C-terminus of ABE increases editing efficiency, similar to what has been observed using C > T base editors. While this observation was not confirmed at all target sites, one possible explanation could be that the UGI prevents removal of the hypoxanthine created by the adenine deaminase. Should this explanation prove true, it emphasizes the importance of manipulating the DNA repair pathways to achieve improved editing rates. These findings also suggest that further improvements can potentially be accomplished by fusing ABE to other protein domains that regulate DNA repair.

Other groups have recently accomplished improved modification rates using C > T base editors by implementing codon optimization algorithms that removed potential polyadenylation sequences from the Cas9 open reading frame as well as adding additional nuclear localization signals^38^. The ABE that we used in these experiments has been codon optimized and we did not identify any polyadenylation signals within Cas9; however, inclusion of additional nuclear localization signals could improve further the editing efficiency, which might be particularly important for therapeutic applications requiring high levels of correction.

Adenine base editors were used recently in vivo by AAV delivery to correct a mutation that causes DMD in the *mdx* mouse model^39^. However, this work was performed using a dual trans-splicing AAV system, which relies on ITR homology and whose efficiency is typically considered limited. While we have not performed a direct comparison between this approach and the intein-based split system that we developed, it is noteworthy that our split base editor architecture appears to be at least as efficient as ABE 7.10 in vitro. This result was unexpected because the intein system requires assembly of the full-length ABE and excision of the intein after translation and before editing can occur and, therefore, the kinetics of the reaction are less favorable than that of ABE 7.10. However, it is possible that expression of two shorter transcripts from two independent promoters increases the efficiency of transcription and translation of ABE, thus offsetting a potentially slower editing reaction. It remains to be determined whether this system is also efficient in vivo after systemic AAV administration, although the data from Ryu et al.^39^ supports that reconstitution in vivo after delivery from two different viral particles is possible.

In summary, the ability to disrupt splice acceptor sites using adenine base editors further expands the available tools for inducing therapeutic exon skipping. It proves especially useful for exon targets that are not accessible by BE3 due to PAM restrictions and further increases the total amount of exons that can be skipped using single-base editors. In addition, when both ABE and BE3 can be used to target the same exon, ABE generally offers higher on-target activity and lower off-target activity. These improved ABE variants and the split ABE architecture that we developed represent significant progress towards enabling in vivo base editing studies since the potential to package a base editor into AAV particles is an advancement that will enable exploration of various therapies, which will have a lasting impact on the field of gene therapy.

## Materials and methods

### Plasmids and cloning

The plasmid used for SpCas9 sgRNA expression and expression of SpCas9, dCas9, and Cas9-D10A were gifts from Charles Gersbach. The ABE 7.10 plasmid was generated through Gibson assembly of a gBlock Gene Fragment (Integrated DNA Technologies) containing the TadA domains and ABE 7.10 linker, as described by Gaudelli et al.^13^, into a Cas9-D10A backbone (Addgene plasmid #41816). The ABE plasmids containing the various linkers described in Fig. 2A were created through Gibson assembly of gBlock Gene Fragments into the ABE 7.10 plasmid (Supplemental Sequences). The ABE-UGI plasmid was generated through Gibson assembly of the TadA deaminase domains into an spCas9-BE3 plasmid (pCMV-BE3) that was a gift from David Liu (Addgene plasmid #73021). Split-ABE constructs were generated through Gibson assembly of gBlock Gene Fragments. Amino acid sequences are provided in Supplemental Sequences. All base editor constructs were under the control of the CMV promoter, except for N-ABE-AAV which was under the control of an EFS promoter^40^.

All oligonucleotides used in this work were obtained from IDT. The oligonucleotides for sgRNA generation were hybridized, phosphorylated and cloned into the SpCas9 sgRNA vector using BbsI sites^41,42^. Guide sequences are provided in Supplementary Table S4. All sgRNA cassettes were under control of the human U6 promoter.

### Cell culture and transfection

HEK293T, HCT116, Neuro2A, and Hepa1–6 cell lines were obtained from the American Tissue Collection Center (ATCC). They were maintained in DMEM supplemented with 10% fetal bovine serum and 1% penicillin/streptomycin at 37 °C with 5% CO_2_. Transfections were performed in 24-well plates using Lipofectamine 2000 (Invitrogen) following manufacturer’s instructions. The amount of DNA used for lipofection was 1 µg per well. Transfection efficiency was routinely higher than 80% as determined by fluorescent microscopy following delivery of a control GFP-expression plasmid.

### AAV vector production

HEK293T cells were seeded in 15 cm dishes and transfected at 80–90% confluence. GFP-AAV plasmid, N-ABE-AAV or C-ABE-AAV were transfected along with pHelper and pAAV-DJ from the AAV-DJ Packaging System from Cell Biolabs in a 1:1:1 ratio using calcium phosphate and a total of 60 µg per plate. Media was replaced 24 h post transfection. Cell pellets were harvested at 72 h post transfection through manual cell scraping and centrifuged at 1500×*g* for 12 min. After aspirating the supernatant, the cell pellet was resuspended in 1 mL AAV lysis buffer (50 mM Tris-HCl pH = 8.5, 150 mM NaCl and 2 mM MgCl_2_). Resuspended pellets were subjected to three freeze–thaw cycles between an ethanol/dry ice bath and a 37 °C water bath. Lysed cell pellets were then spun at 10,000×*g* for 10 min and the supernatant was collected as crude lysate. Lysates were then treated with 50 U benzonase per mL and incubated at 37 °C for 30 min to digest unpackaged plasmid. Crude lysates were added directly to cells or flash frozen with liquid nitrogen and stored at −80 °C for future use.

### AAV infection

HEK293T cells were infected in suspension in the wells of a 24-well plate by mixing 100 µL of crude lysate with 20,000 cells in 150 µL of cell culture medium. In the case of the samples containing both N-ABE AAV and C-ABE AAV, 50 µL of each lysate was added. Protamine sulfate was added to the lysate-cell mix at a final concentration of 5 µg/mL to enhance infection efficiency. Cells were incubated for 24 h at which point the media was aspirated and replaced with 500 µL of fresh medium. Infected cells were incubated for a total of 6 days before harvesting genomic DNA and RNA for analysis.

### RT-PCR

RNA was harvested from cell pellets using the RNeasy Plus Mini Kit (Qiagen) according to manufacturer’s instructions. cDNA synthesis was performed using the qScript cDNA Synthesis Kit (Quanta Biosciences) from 500–1000 ng of RNA with cycling conditions performed as directed by the supplier. PCR was performed using KAPA2G Robust PCR kits from Kapa Biosystems. The 25 µL reactions used 50 ng of cDNA, Buffer A (5 µL), Enhancer (5 µL), dNTPs (0.5 µL), 10 µM forward primer (1.25 µL), 10 µM reverse primer (1.25 µL), KAPA2G Robust DNA Polymerase (0.5 U), and water (up to 25 µL). We used cycling parameters as recommended by the manufacturer. The PCR products were visualized in 2% agarose gels stained with ethidium bromide and images were captured using a ChemiDoc-It2 (UVP). The DNA sequences of the primers for each target are provided in Supplementary Table S5.

### Densitometry analysis

Skipping efficiencies were determined by densitometry analysis of the PCR products obtained from RT-PCR and analyzed by agarose gel electrophoresis using ImageJ software. After subtracting background noise, band intensity was compared using the following formula: $\% \;\mathrm{Exon}\;\mathrm{skipping} = \frac{\mathrm{Skipped}\;\mathrm{band}\;\mathrm{intensity}}{\mathrm{wt}\;\mathrm{Band}\;\mathrm{intensity} + \mathrm{Skipped}\;\mathrm{band}\;\mathrm{intensity}},$ where band intensity is the sum of each pixel grayscale value within the selected area of the band.

### Amplification of genomic DNA

Genomic DNA was isolated using a DNeasy Blood and Tissue Kit (Qiagen) and PCR amplification was performed with KAPA2G Robust PCR kits (KAPA Biosystems) as described above, using 20–100 ng of template DNA.

### Editing window analysis using Sanger sequencing and EditR software

Genomic DNA from samples treated with an ABE-GGGGS variant and an A-rich sgRNA was amplified using the PCR primers listed in Supplementary Table S5. Sanger sequencing of the PCR amplicons was performed by the W.M. Keck Center for Comparative and Functional Genomics at the University of Illinois at Urbana-Champaign using the primers listed in Supplementary Table S6. Base editing efficiencies were estimated by analyzing the sanger sequencing traces using EditR^43^.

### High-throughput sequencing

HTS was performed on PCR amplicons from genomic DNA or RNA harvested from duplicate transfections of HEK293T cells. PCR primers for HTS are provided in Supplementary Table S7. After validating the quality of PCR products by gel electrophoresis the PCR products were isolated by gel extraction using the Zymoclean Gel DNA Recovery Kit (Zymo Research). Indexed HTS amplicon libraries for samples described in Figs. 4 and 5 were prepared with the Hyper Library construction kit from Kapa Biosystems without shearing. Indexed HTS amplicon libraries for samples described in Figs. 6 and 7 were prepared using a Nextera XT DNA Library Prep Kit (Illumina). The libraries were quantitated by qPCR and sequenced on MiSeq Nano flowcells for 251 cycles from each end of the fragments using a MiSeq 500-cycle sequencing kit version 2. Fastq files were generated and demultiplexed with the bcl2fastq v2.17.1.14 Conversion Software (Illumina). All sequencing was performed by the W.M. Keck Center for Comparative and Functional Genomics at the University of Illinois at Urbana-Champaign. All sequencing data are available at Gene Expression Omnibus under Series GSE120125.

### Sequence analysis

DNA and RNA sequencing reads were demultiplexed by PCR primer sequences and quality trimmed to Phred quality score 20 at the 3′ end using cutadapt. Read pairs with at least one mate trimmed to 50 bp or less were discarded. DNA reads were then aligned to the human genome version GRCh38 using Bowtie2^44^. To determine on-target and off-target base editing rates, alternative allele depths were calculated by Samtools mpileup over 120 bp windows centered around the protospacer sequences for on and off targets. A global estimate of sequencing error was made by averaging the fraction of alternative allele depths across all positions. A position-dependent estimate of sequencing error was determined by fraction of alternative allele depth at each genomic position. Significant A > G or T > C conversion was determined by using the one-sided binomial test at a *p* value cutoff of 10^−5^, using the higher of the global or position-dependent sequencing error estimates as the background probability of nucleotide conversions. Indel rates were calculated using Mutect2^45^.

Reads from paired-end RNA-seq were mapped to the human genome version GRCh38 with TopHat2^46^ for isoform quantification. Forward and reverse reads were combined as a single read for analysis. Reads displaying the exon-skipped junction were counted toward the exon-skipped transcript and reads displaying either the 5′ or 3′ canonical splice junction were counted towards the canonical isoform. Reads that did not display any of the previously mentioned splice junctions were excluded from quantification. The exon-skipping rate for each biological duplicate was calculated by dividing the number of exon-skipped transcript reads by the sum of the number of exon-skipped and canonical transcript reads. Estimates of the overall exon-skipping rates were made by averaging duplicates.

### Genome-wide targetability analysis

All exons of protein coding transcripts (genomic assembly GRCh38, GENCODE release 26) that are not the first or last exon in a transcript were scanned for PAMs in the appropriate range. The predicted position-dependent base editing efficiencies for BE3 are identical to those used in Gapinske et al.^14^. The corresponding efficiency values for ABE were estimated from ABE7.8 efficiency values from Gaudelli et al.^13^, Fig. 3c and Supplemental Fig. S7b by the following method: first, the maximum base editing efficiency was estimated by taking the highest observed editing efficiency across all ABE variants and sites from Fig. 3c; then, the relative base editing efficiencies of ABE7.8 from Supplemental Fig. S7b positions 4–9 were multiplied by the estimated maximum base editing efficiency to obtain the estimated position-dependent base editing efficiencies for ABE.

For each candidate sgRNA, the entire genome was scanned for all sequences with at most two mismatches and an off-target score was calculated^34^. Any sgRNA with an off-target score above ten was removed.

## Supplementary information

Supplemental Material

## Supplementary information

**Supplementary Information** accompanies the paper at (10.1038/s41421-019-0109-7).

## References

[1] Pan Q, Shai O, Lee LJ, Frey BJ, Blencowe BJ (2008). Deep surveying of alternative splicing complexity in the human transcriptome by high-throughput sequencing. Nat. Genet..

[2] Kole R, Krieg AM (2015). Exon skipping therapy for Duchenne muscular dystrophy. Adv. Drug Deliv. Rev..

[3] Kowalewski C (2016). Amelioration of junctional epidermolysis bullosa due to exon skipping. Br. J. Dermatol..

[4] Burghes AH, McGovern VL (2010). Antisense oligonucleotides and spinal muscular atrophy: skipping along. Genes Dev..

[5] Crooke ST (1999). Molecular mechanisms of action of antisense drugs. Biochim. Biophys. Acta.

[6] Mou H (2017). CRISPR/Cas9-mediated genome editing induces exon skipping by alternative splicing or exon deletion. Genome Biol..

[7] Long C (2018). Correction of diverse muscular dystrophy mutations in human engineered heart muscle by single-site genome editing. Sci. Adv..

[8] Ceccaldi R, Rondinelli B, D’Andrea AD (2016). Repair pathway choices and consequences at the double-strand break. Trends Cell Biol..

[9] Haapaniemi E, Botla S, Persson J, Schmierer B, Taipale J (2018). CRISPR-Cas9 genome editing induces a p53-mediated DNA damage response. Nat. Med..

[10] Ihry RJ (2018). p53 inhibits CRISPR-Cas9 engineering in human pluripotent stem cells. Nat. Med..

[11] Kosicki M, Tomberg K, Bradley A (2018). Repair of double-strand breaks induced by CRISPR-Cas9 leads to large deletions and complex rearrangements. Nat. Biotechnol..

[12] Komor AC, Kim YB, Packer MS, Zuris JA, Liu DR (2016). Programmable editing of a target base in genomic DNA without double-stranded DNA cleavage. Nature.

[13] Gaudelli NM (2017). Programmable base editing of A*T to G*C in genomic DNA without DNA cleavage. Nature.

[14] Gapinske M (2018). CRISPR-SKIP: programmable gene splicing with single base editors. Genome Biol..

[15] Yuan J (2018). Genetic modulation of rna splicing with a crispr-guided cytidine deaminase. Mol. Cell.

[16] Sibley CR, Blazquez L, Ule J (2016). Lessons from non-canonical splicing. Nat. Rev. Genet..

[17] Gerard X (2012). AON-mediated exon skipping restores ciliation in fibroblasts harboring the common leber congenital amaurosis cep290 mutation. Mol. Ther. Nucleic Acids.

[18] Khoo B, Roca X, Chew SL, Krainer AR (2007). Antisense oligonucleotide-induced alternative splicing of the APOB mRNA generates a novel isoform of APOB. BMC Mol. Biol..

[19] Kalbfuss B, Mabon SA, Misteli T (2001). Correction of alternative splicing of Tau in frontotemporal dementia and Parkinsonism linked to chromosome 17. J. Biol. Chem..

[20] Mercatante DR, Mohler JL, Kole R (2002). Cellular response to an antisense-mediated shift of Bcl-x pre-mRNA splicing and antineoplastic agents. J. Biol. Chem..

[21] Karras JG, McKay RA, Dean NM, Monia BP (2000). Deletion of individual exons and induction of soluble murine interleukin-5 receptor-alpha chain expression through antisense oligonucleotide-mediated redirection of pre-mRNA splicing. Mol. Pharmacol..

[22] Goto M (2006). Targeted skipping of a single exon harboring a premature termination codon mutation: implications and potential for gene correction therapy for selective dystrophic epidermolysis bullosa patients. J. Invest. Dermatol..

[23] Aartsma-Rus A, van Ommen GJB (2007). Antisense-mediated exon skipping: a versatile tool with therapeutic and research applications. RNA.

[24] van Putten M (2013). Low dystrophin levels increase survival and improve muscle pathology and function in dystrophin/utrophin double-knockout mice. FASEB J..

[25] Dawson TM, Li D, Yue Y, Duan D (2010). Marginal level dystrophin expression improves clinical outcome in a strain of dystrophin/utrophin double knockout mice. PLoS ONE.

[26] Chen X, Zaro JL, Shen WC (2013). Fusion protein linkers: property, design and functionality. Adv. Drug Deliv. Rev..

[27] Lee HW, Dominy BN, Cao W (2011). New family of deamination repair enzymes in uracil-DNA glycosylase superfamily. J. Biol. Chem..

[28] Daya S, Berns KI (2008). Gene therapy using adeno-associated virus vectors. Clin. Microbiol. Rev..

[29] Penaud-Budloo M (2008). Adeno-associated virus vector genomes persist as episomal chromatin in primate muscle. J. Virol..

[30] Wu Z, Yang H, Colosi P (2010). Effect of genome size on AAV vector packaging. Mol. Ther..

[31] Chew WL (2016). A multifunctional AAV–CRISPR–Cas9 and its host response. Nat. Methods.

[32] Hsu PD (2013). DNA targeting specificity of RNA-guided Cas9 nucleases. Nat. Biotechnol..

[33] Fu Y (2013). High-frequency off-target mutagenesis induced by CRISPR-Cas nucleases in human cells. Nat. Biotechnol..

[34] Kwak H, Fuda NJ, Core LJ, Lis JT (2013). Precise maps of RNA polymerase reveal how promoters direct initiation and pausing. Science.

[35] Fuchs G (2014). 4sUDRB-seq: measuring genomewide transcriptional elongation rates and initiation frequencies within cells. Genome Biol..

[36] Veloso A (2014). Rate of elongation by RNA polymerase II is associated with specific gene features and epigenetic modifications. Genome Res..

[37] Alexander RD, Innocente SA, Barrass JD, Beggs JD (2010). Splicing-dependent RNA polymerase pausing in yeast. Mol. Cell.

[38] Zafra MP (2018). Optimized baseeditors enable efficient editing in cells, organoids and mice. Nat. Biotechnol..

[39] Ryu SM (2018). Adenine base editing in mouse embryos and an adult mouse model of Duchenne muscular dystrophy. Nat. Biotechnol..

[40] Tabebordbar M (2016). In vivo gene editing in dystrophic mouse muscle and muscle stem cells. Science.

[41] Brown A, Woods WS, Perez-Pinera P (2016). Multiplexed targeted genome engineering using a universal nuclease-assisted vector integration system. ACS Synth. Biol..

[42] Gapinske M, Tague N, Winter J, Underhill GH, Perez-Pinera P (2018). Targeted gene knock out using nuclease-assisted vector integration: hemi- and homozygous deletion of JAG1. Methods Mol. Biol.

[43] Kluesner MG (2018). EditR: a method to quantify base editing from Sanger sequencing. CRISPR J..

[44] Langmead B, Salzberg SL (2012). Fast gapped-read alignment with Bowtie 2. Nat. Methods.

[45] Cibulskis K (2013). Sensitive detection of somatic point mutations in impure and heterogeneous cancer samples. Nat. Biotechnol..

[46] Kim D (2013). TopHat2: accurate alignment of transcriptomes in the presence of insertions, deletions and gene fusions. Genome Biol..

