# Author Correction: Targeted exon skipping with AAV-mediated split adenine base editors

**DOI:** 10.1038/s41421-019-0125-7

**Published:** 2019-10-15

**Authors:** Jackson Winter, Alan Luu, Michael Gapinske, Sony Manandhar, Shraddha Shirguppe, Wendy S. Woods, Jun S. Song, Pablo Perez-Pinera

**Affiliations:** 1 Department of Bioengineering, University of Illinois at Urbana-Champaign, Urbana, IL 61801 USA; 2 Department of Physics, University of Illinois at Urbana-Champaign, Urbana, IL 61801 USA; 3 Carl R. Woese Institute for Genomic Biology, University of Illinois at Urbana-Champaign, Urbana, IL 61801 USA; 4 Carle Illinois College of Medicine, Champaign, IL 61820 USA; 5 Cancer Center at Illinois, University of Illinois at Urbana-Champaign, Urbana, IL 61801 USA

**Correction to: Cell Discovery (2019) 5:41**

10.1038/s41421-019-0109-7, Published online 20 August 2019

In the original publication of this article^1^, Fig. 4 was labelled incorrectly. The second panel displaying *HSF1* Exon 10 should be labelled as *HSF1* Exon 11, while the third panel displaying *JUP* Exon 11 should be labelled as *JUP* Exon 10. The same labelling change should be applied to the corresponding panels in Fig. 5, as *HSF1* Exon 10 and JUP Exon 11 were incorrectly labelled in that figure as well and should be replaced with *HSF1* Exon 11 and *JUP* Exon 10, respectively.

**Fig. 4:**
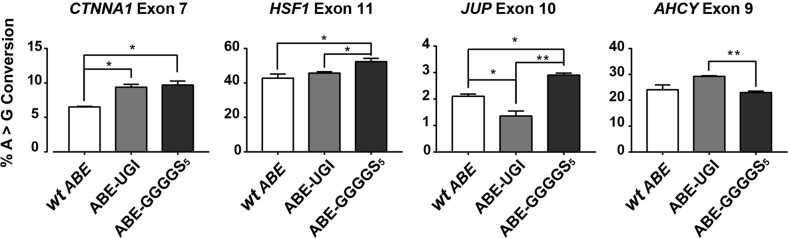
Quantification of genomic DNA mutation rates created by several ABE constructs at multiple target sites. High-throughput sequencing was used to quantify rates of A > G genomic DNA mutation and rates of exon skipping across multiple targets using several ABE variants. * and ** correspond to *P* < 0.05 and *P* < 0.01, respectively by two-tailed unpaired Student’s *t* test across two biological replicates

**Fig. 5:**
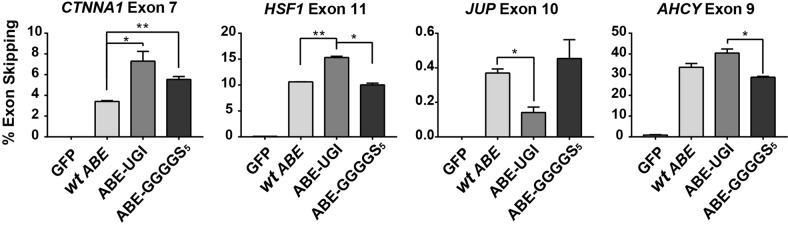
Quantification of exon skipping rates at multiple gene targets induced by several ABE constructs. High-throughput sequencing of cDNA was used to quantify rates of exon skipping across multiple targets using several ABE variants. * and ** correspond to *P* < 0.05 and *P* < 0.01, respectively by two-tailed unpaired Student’s *t* test across two biological replicates

All instances of *HSF1* exon 10 within the text should be corrected to *HSF1* exon 11 and all instances of *JUP* exon 11 should be corrected to JUP exon 10.
