# Supplementary material for: Targeted exon skipping with AAV-mediated split adenine base editors
Source: Cell Discov. 2019 Aug 20;5:41. doi: 10.1038/s41421-019-0109-7 (PMC6796986; doi:10.1038/s41421-019-0109-7)
Supplement: Supplementary file 1 — Supplemental Material [file 41421_2019_109_MOESM1_ESM.pdf]

Supplementary Fig. S1

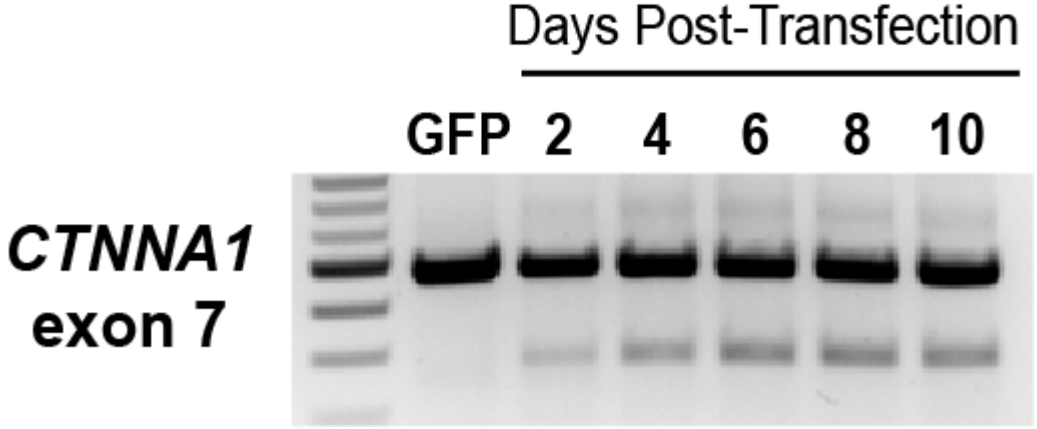

Supplementary Fig. S1: RT-PCR was used to detect exon skipping over a 10 day time course. Maximal levels of skipping were observed 6 days post-transfection.

Supplementary Fig. S2

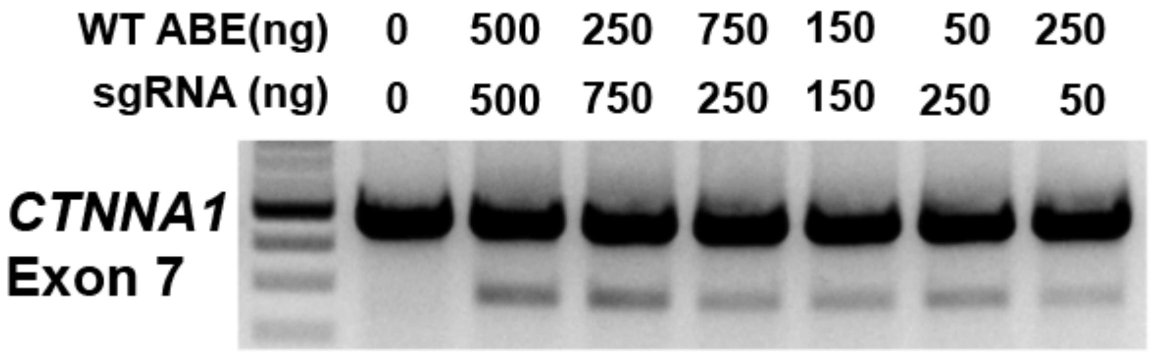

Supplementary Fig. S2: RT-PCR following transfection of HEK293T cells with various concentrations of plasmids encoding ABE and a sgRNA targeting exon 7 of the *CTNNA1* gene.

Supplementary Fig. S3

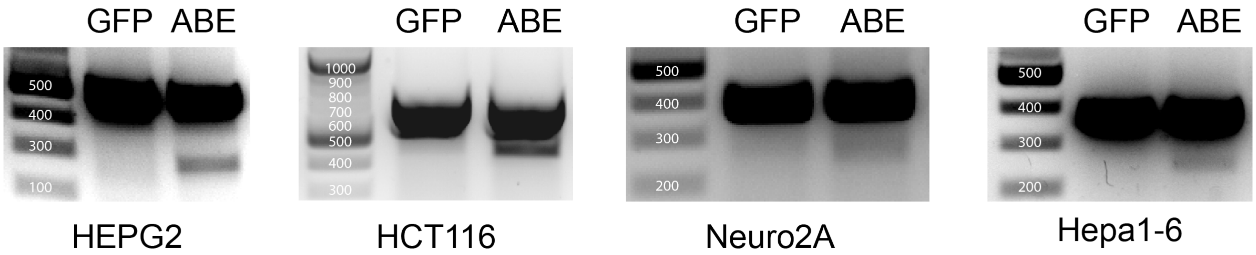

Supplementary Fig. S3: RT-PCR products demonstrating targeted exon skipping of *CTNNA1* exon 7 in HEPG2 cells, *AHCY* exon 9 in HCT116 cells, and *CTNNB1* in mouse Neruro2A and mouse Hepa1-6 cells. PCR primers and expected amplicon sizes are listed in **Supplementary Table S5**.

Supplementary Fig. S4

Target 1

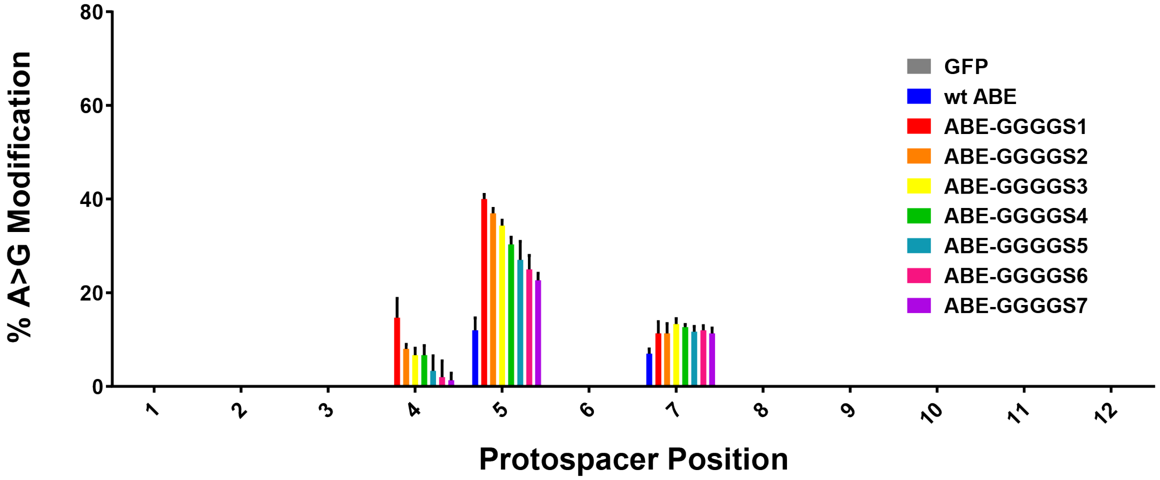

Target 2

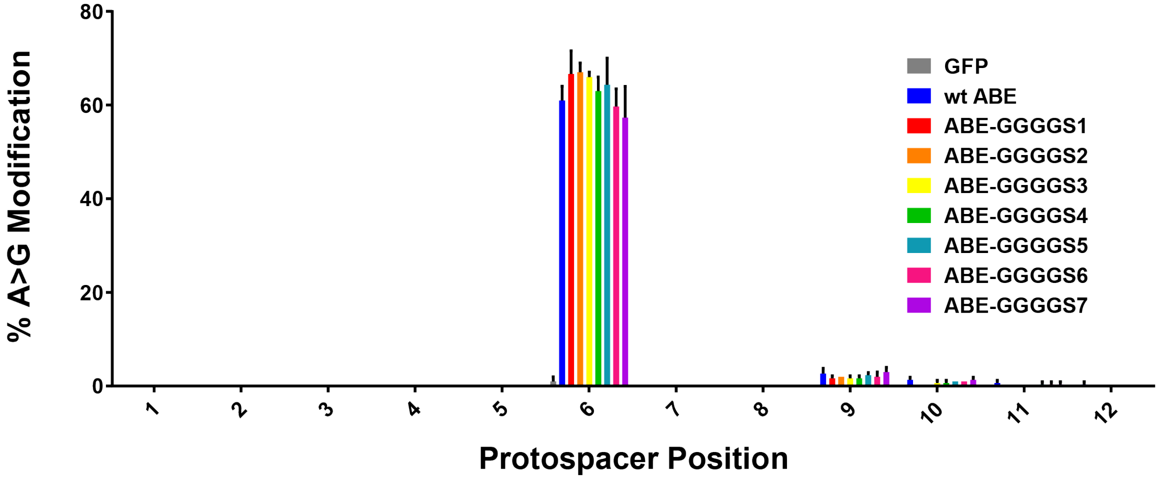

Supplementary Fig. S4: Estimates of positional A>G modification efficiencies at each of the target A's within the protospacer of A-Rich Target 1 and A-Rich Target 2 using EditR software (n = 3). Position 1 represents the base farthest from the start of the PAM using a 20bp sgRNA. ABE-GGGGS constructs enabled editing of position 4, which was not observed with *wt* ABE. Additionally, shorter linker lengths corresponded to higher editing rates for positions 4 and 5, with ABE-GGGGS1 achieving the highest rates of base editing.

Supplementary Fig. S5

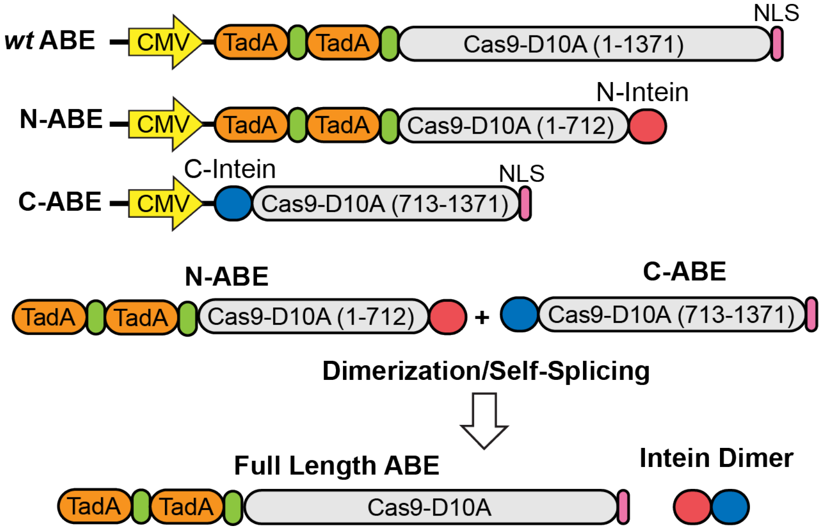

Supplementary Fig. S5: Schematic representation of the split-ABE plasmid system. N-terminal and C-terminal intein sequences reconstitute the full-length protein when co-expressed within the cell.

Supplementary Fig. S6

GFP	+	-	-	-	-
sgRNA	-	+	+	+	+
wt ABE	-	+	-	-	-
N-ABE	-	-	+	-	+
C-ABE	-	-	-	+	+

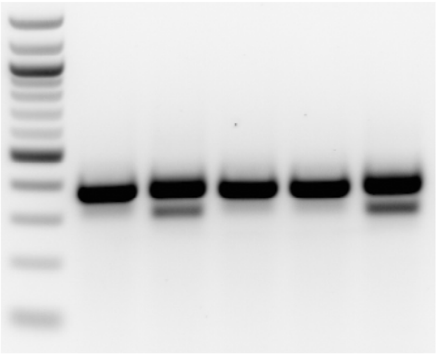

Supplementary Fig. S6: HEK293T cells were transfected with either GFP or a combination of sgRNA targeting AHCY exon 9 and N-ABE, C-ABE or both and their RNA was used in RT-PCR to detect targeted exon skipping of *HSF1* exon 11. Only when both split ABE plasmids are present we were able to detect exon skipping.

Supplementary Fig. S7

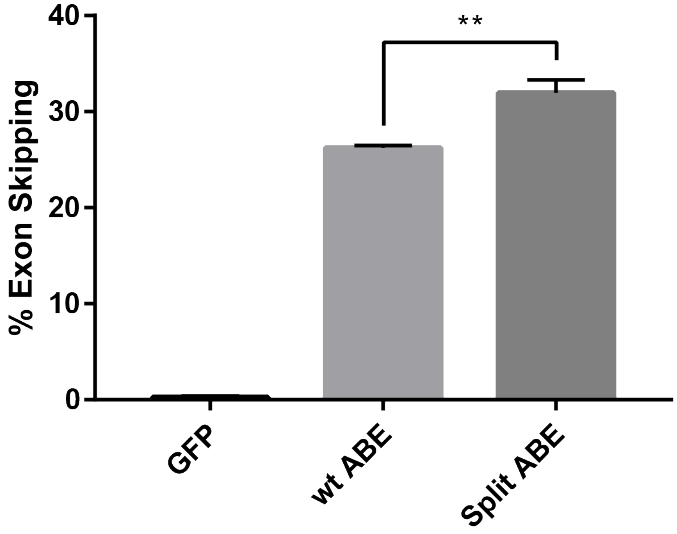

Supplementary Fig. S7: High throughput sequencing analysis of RT-PCR products demonstrated significantly increased levels of exon skipping by the split ABE system at 32.0% compared to 26.2% with the full length *wt* ABE (*P* = .002 by two-tailed unpaired Students t-test (n = 3).

Supplementary Fig. S8

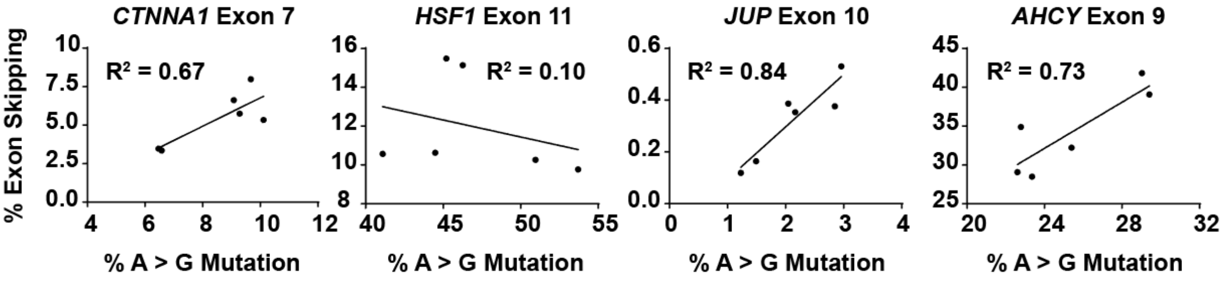

Supplementary Fig. S8: Rates of exon skipping showed a linear co-relation with rates of A>G mutations within splice acceptor sites for most targets tested.

GFP Control	*CTNNA1* Intron 7							*CTNNA1* Exon 7													PAM			% Indel
CTNNA1 Exon 7	T1	C2	T3	G4	C5	A6	G7	A8	A9	A10	C11	A12	A13	A14	T15	C16	A17	T18	T19	G20	T	G	G	0.00%
A	0.1%	0.0%	0.0%	0.1%	0.1%	99.7%	0.1%	99.7%	99.5%	99.5%	0.1%	99.7%	99.8%	99.7%	0.0%	0.1%	99.8%	0.0%	0.3%	0.1%	0.1%	0.1%	0.2%	
C	0.2%	99.9%	0.2%	0.0%	99.8%	0.0%	0.0%	0.0%	0.1%	0.1%	99.7%	0.1%	0.0%	0.1%	0.1%	99.8%	0.0%	0.2%	0.6%	0.1%	0.2%	0.0%	0.2%	
G	0.2%	0.0%	0.1%	99.8%	0.0%	0.3%	99.9%	0.2%	0.3%	0.3%	0.1%	0.2%	0.2%	0.2%	0.1%	0.0%	0.2%	0.1%	0.2%	99.5%	0.1%	99.8%	99.3%	
T	99.5%	0.0%	99.7%	0.1%	0.1%	0.0%	0.1%	0.0%	0.1%	0.1%	0.1%	0.0%	0.0%	0.0%	99.7%	0.1%	0.0%	99.7%	98.8%	0.2%	99.6%	0.1%	0.2%	

WT ABE																								% Indel
CTNNA1 Exon 7	T1	C2	T3	G4	C5	A6	G7	A8	A9	A10	C11	A12	A13	A14	T15	C16	A17	T18	T19	G20	T	G	G	0.66%
A	0.1%	0.0%	0.0%	0.1%	0.0%	93.4%	0.1%	99.5%	99.4%	99.4%	0.1%	99.6%	99.7%	99.7%	0.0%	0.1%	99.7%	0.0%	0.3%	0.2%	0.1%	0.1%	0.2%	
C	0.3%	99.9%	0.3%	0.0%	99.8%	0.0%	0.0%	0.0%	0.1%	0.1%	99.7%	0.1%	0.0%	0.1%	0.2%	99.8%	0.0%	0.2%	0.7%	0.1%	0.3%	0.0%	0.2%	
G	0.2%	0.0%	0.1%	99.8%	0.0%	6.5%	99.9%	0.5%	0.4%	0.4%	0.1%	0.3%	0.2%	0.2%	0.1%	0.0%	0.2%	0.1%	0.1%	99.6%	0.1%	99.8%	99.5%	
T	99.5%	0.1%	99.6%	0.0%	0.1%	0.0%	0.1%	0.0%	0.1%	0.1%	0.1%	0.0%	0.0%	0.0%	99.6%	0.1%	0.0%	99.7%	98.9%	0.2%	99.5%	0.1%	0.2%	

ABE-UGI																								% Indel
CTNNA1 Exon 7	T1	C2	T3	G4	C5	A6	G7	A8	A9	A10	C11	A12	A13	A14	T15	C16	A17	T18	T19	G20	T	G	G	0.00%
A	0.1%	0.0%	0.0%	0.1%	0.0%	90.5%	0.1%	99.4%	99.4%	99.4%	0.1%	99.6%	99.7%	99.6%	0.1%	0.1%	99.7%	0.0%	0.3%	0.1%	0.1%	0.1%	0.2%	
C	0.3%	99.9%	0.3%	0.0%	99.8%	0.0%	0.0%	0.0%	0.1%	0.1%	99.7%	0.1%	0.0%	0.1%	0.3%	99.8%	0.0%	0.2%	0.7%	0.1%	0.2%	0.0%	0.2%	
G	0.2%	0.0%	0.1%	99.8%	0.0%	9.4%	99.9%	0.5%	0.4%	0.5%	0.1%	0.3%	0.3%	0.3%	0.1%	0.0%	0.2%	0.1%	0.2%	99.6%	0.1%	99.8%	99.4%	
T	99.5%	0.0%	99.6%	0.1%	0.1%	0.0%	0.1%	0.0%	0.1%	0.1%	0.1%	0.0%	0.0%	0.1%	99.6%	0.1%	0.0%	99.7%	98.8%	0.2%	99.6%	0.1%	0.2%	

ABE-GGGGS5																								% Indel
CTNNA1 Exon 7	T1	C2	T3	G4	C5	A6	G7	A8	A9	A10	C11	A12	A13	A14	T15	C16	A17	T18	T19	G20	T	G	G	0.00%
A	0.1%	0.0%	0.0%	0.1%	0.1%	90.2%	0.1%	99.4%	99.3%	99.3%	0.1%	99.6%	99.7%	99.5%	0.0%	0.1%	99.7%	0.0%	0.3%	0.2%	0.1%	0.1%	0.3%	
C	0.3%	99.9%	0.3%	0.0%	99.8%	0.0%	0.0%	0.1%	0.1%	0.2%	99.6%	0.1%	0.0%	0.1%	0.2%	99.7%	0.0%	0.2%	0.6%	0.1%	0.3%	0.0%	0.3%	
G	0.3%	0.0%	0.2%	99.7%	0.0%	9.7%	99.8%	0.5%	0.5%	0.4%	0.1%	0.3%	0.3%	0.3%	0.2%	0.0%	0.2%	0.1%	0.3%	99.5%	0.1%	99.7%	99.2%	
T	99.3%	0.1%	99.5%	0.1%	0.1%	0.0%	0.1%	0.1%	0.1%	0.1%	0.1%	0.0%	0.0%	0.1%	99.5%	0.1%	0.0%	99.6%	98.8%	0.2%	99.5%	0.1%	0.2%	

GFP Control	*HSF1* Intron 11							*HSF1* Exon 11													PAM			% Indel
HSF1 Exon 11	T1	C2	C3	G4	C5	A6	G7	C8	T9	G10	T11	T12	C13	A14	G15	C16	C17	C18	C19	T20	C	G	G	0.00%
A	0.4%	0.0%	0.0%	0.1%	0.1%	96.6%	0.2%	0.1%	0.1%	0.2%	0.1%	0.1%	0.1%	97.3%	0.1%	0.0%	0.1%	0.0%	0.1%	0.2%	0.1%	0.2%	0.1%	
C	5.5%	99.9%	99.8%	0.8%	99.7%	1.6%	0.6%	99.7%	0.7%	0.2%	0.5%	1.4%	99.7%	1.3%	0.6%	99.8%	99.8%	99.8%	99.7%	1.6%	99.7%	0.1%	0.1%	
G	1.6%	0.0%	0.1%	98.8%	0.1%	1.4%	98.9%	0.1%	1.2%	98.9%	0.7%	0.9%	0.1%	1.2%	99.1%	0.0%	0.0%	0.0%	0.1%	1.8%	0.1%	99.7%	99.6%	
T	92.5%	0.0%	0.1%	0.3%	0.1%	0.4%	0.4%	0.1%	98.0%	0.7%	98.7%	97.6%	0.1%	0.1%	0.2%	0.1%	0.1%	0.1%	0.2%	96.3%	0.1%	0.1%	0.3%	

WT ABE																								% Indel
HSF1 Exon 11	T1	C2	C3	G4	C5	A6	G7	C8	T9	G10	T11	T12	C13	A14	G15	C16	C17	C18	C19	T20	C	G	G	0.00%
A	0.3%	0.0%	0.0%	0.1%	0.1%	55.3%	0.1%	0.1%	0.1%	0.2%	0.0%	0.2%	0.1%	97.3%	0.2%	0.0%	0.0%	0.0%	0.1%	0.2%	0.0%	0.1%	0.1%	
C	5.8%	99.9%	99.7%	0.7%	99.7%	1.1%	0.5%	99.7%	0.6%	0.2%	0.6%	1.4%	99.8%	1.3%	0.6%	99.9%	99.8%	99.8%	99.8%	1.5%	99.7%	0.0%	0.1%	
G	1.7%	0.0%	0.1%	98.9%	0.1%	43.3%	99.1%	0.1%	1.3%	99.1%	0.7%	0.8%	0.1%	1.2%	99.1%	0.0%	0.0%	0.1%	0.1%	1.6%	0.1%	99.7%	99.6%	
T	92.2%	0.0%	0.1%	0.3%	0.1%	0.4%	0.3%	0.2%	98.0%	0.6%	98.7%	97.6%	0.1%	0.1%	0.2%	0.1%	0.1%	0.1%	0.1%	96.7%	0.1%	0.1%	0.2%	

ABE-UGI																								% Indel
HSF1 Exon 11	T1	C2	C3	G4	C5	A6	G7	C8	T9	G10	T11	T12	C13	A14	G15	C16	C17	C18	C19	T20	C	G	G	0.00%
A	0.3%	0.0%	0.1%	0.1%	0.1%	52.4%	0.2%	0.1%	0.1%	0.2%	0.1%	0.1%	0.1%	97.3%	0.1%	0.1%	0.1%	0.1%	0.1%	0.2%	0.0%	0.1%	0.1%	
C	5.7%	99.9%	99.7%	0.8%	99.7%	1.0%	0.6%	99.7%	0.7%	0.2%	0.6%	1.5%	99.7%	1.3%	0.7%	99.8%	99.8%	99.8%	99.7%	1.6%	99.7%	0.1%	0.1%	
G	1.8%	0.0%	0.1%	98.8%	0.1%	46.2%	98.9%	0.1%	1.4%	98.9%	0.8%	0.9%	0.1%	1.2%	99.0%	0.0%	0.0%	0.1%	0.1%	1.6%	0.1%	99.7%	99.5%	
T	92.2%	0.0%	0.1%	0.3%	0.1%	0.4%	0.4%	0.1%	97.8%	0.7%	98.6%	97.5%	0.1%	0.1%	0.2%	0.1%	0.1%	0.1%	0.1%	96.6%	0.1%	0.2%	0.3%	

ABE-GGGGS5																								% Indel
HSF1 Exon 11	T1	C2	C3	G4	C5	A6	G7	C8	T9	G10	T11	T12	C13	A14	G15	C16	C17	C18	C19	T20	C	G	G	0.00%
A	0.4%	0.1%	0.1%	0.1%	0.1%	46.1%	0.2%	0.1%	0.1%	0.2%	0.3%	0.3%	0.1%	97.0%	0.1%	0.1%	0.1%	0.1%	0.1%	0.2%	0.0%	0.1%	0.1%	
C	6.1%	99.8%	99.7%	0.8%	99.3%	1.1%	0.7%	99.6%	0.8%	0.3%	0.6%	1.6%	99.7%	1.5%	0.7%	99.8%	99.8%	99.7%	99.7%	1.7%	99.7%	0.1%	0.1%	
G	2.0%	0.1%	0.2%	98.7%	0.2%	52.4%	98.8%	0.1%	1.5%	98.8%	0.9%	1.0%	0.1%	1.4%	98.9%	0.0%	0.0%	0.1%	0.1%	1.9%	0.2%	99.6%	99.5%	
T	91.6%	0.0%	0.1%	0.4%	0.4%	0.4%	0.4%	0.2%	97.6%	0.7%	98.2%	97.1%	0.1%	0.1%	0.2%	0.1%	0.1%	0.1%	0.1%	96.2%	0.1%	0.2%	0.3%	

GFP Control	*JUP* Intron 10							*JUP* Exon 10													PAM			% Indel
JUP Exon 10	C1	A2	C3	A4	C5	A6	G7	G8	A9	T10	G11	G12	T13	G14	T15	G16	A17	G18	G19	A20	T	G	G	0.00%
A	0.1%	99.6%	0.1%	99.3%	0.1%	99.6%	0.1%	0.1%	99.2%	0.1%	0.1%	0.1%	0.7%	0.1%	0.3%	0.0%	99.3%	0.0%	0.1%	99.0%	0.1%	0.1%	0.1%	
C	99.9%	0.1%	99.8%	0.3%	99.8%	0.1%	0.0%	0.0%	0.4%	0.4%	0.0%	0.0%	0.9%	0.0%	0.6%	0.0%	0.3%	0.0%	0.0%	0.5%	0.3%	0.0%	0.0%	
G	0.0%	0.2%	0.0%	0.3%	0.0%	0.2%	99.9%	99.8%	0.3%	0.2%	99.8%	99.9%	0.8%	99.9%	0.8%	99.9%	0.3%	99.9%	99.9%	0.3%	0.2%	99.9%	99.8%	
T	0.0%	0.1%	0.1%	0.1%	0.1%	0.0%	0.0%	0.0%	0.1%	99.3%	0.0%	0.0%	97.6%	0.0%	98.3%	0.0%	0.1%	0.0%	0.0%	0.1%	99.5%	0.0%	0.1%	

WT ABE																								% Indel
JUP Exon 10	C1	A2	C3	A4	C5	A6	G7	G8	A9	T10	G11	G12	T13	G14	T15	G16	A17	G18	G19	A20	T	G	G	0.00%
A	0.1%	99.6%	0.0%	99.3%	0.1%	97.8%	0.1%	0.1%	98.6%	0.1%	0.1%	0.1%	0.6%	0.1%	0.2%	0.1%	99.2%	0.1%	0.1%	99.2%	0.0%	0.1%	0.1%	
C	99.9%	0.1%	99.9%	0.3%	99.8%	0.1%	0.0%	0.0%	0.4%	0.4%	0.0%	0.0%	0.9%	0.0%	0.6%	0.0%	0.3%	0.0%	0.0%	0.4%	0.3%	0.0%	0.0%	
G	0.0%	0.3%	0.0%	0.3%	0.0%	2.1%	99.9%	99.9%	0.9%	0.1%	99.9%	99.9%	0.7%	99.9%	0.6%	99.9%	0.4%	99.9%	99.9%	0.3%	0.1%	99.9%	99.9%	
T	0.1%	0.1%	0.1%	0.1%	0.1%	0.1%	0.0%	0.0%	0.1%	99.3%	0.0%	0.0%	97.7%	0.0%	98.5%	0.0%	0.1%	0.0%	0.0%	0.1%	99.6%	0.0%	0.0%	

ABE-UGI																								% Indel
JUP Exon 10	C1	A2	C3	A4	C5	A6	G7	G8	A9	T10	G11	G12	T13	G14	T15	G16	A17	G18	G19	A20	T	G	G	0.00%
A	0.1%	99.6%	0.1%	99.4%	0.1%	98.5%	0.1%	0.1%	98.7%	0.1%	0.1%	0.1%	0.7%	0.1%	0.3%	0.1%	99.1%	0.1%	0.1%	99.0%	0.1%	0.1%	0.1%	
C	99.9%	0.1%	99.8%	0.3%	99.8%	0.1%	0.0%	0.0%	0.5%	0.4%	0.0%	0.0%	0.9%	0.0%	0.6%	0.0%	0.3%	0.0%	0.0%	0.5%	0.3%	0.0%	0.0%	
G	0.0%	0.2%	0.0%	0.3%	0.0%	1.4%	99.9%	99.8%	0.7%	0.1%	99.9%	99.8%	0.8%	99.9%	0.7%	99.9%	0.4%	99.9%	99.9%	0.4%	0.1%	99.9%	99.8%	
T	0.1%	0.1%	0.1%	0.1%	0.1%	0.1%	0.0%	0.1%	0.1%	99.4%	0.0%	0.0%	97.7%	0.0%	98.3%	0.0%	0.1%	0.0%	0.1%	0.1%	99.5%	0.0%	0.1%	

ABE-GGGGS5																								% Indel
JUP Exon 10	C1	A2	C3	A4	C5	A6	G7	G8	A9	T10	G11	G12	T13	G14	T15	G16	A17	G18	G19	A20	T	G	G	0.00%
A	0.1%	99.5%	0.1%	99.2%	0.1%	96.9%	0.1%	0.1%	98.4%	0.2%	0.1%	0.1%	0.8%	0.1%	0.4%	0.1%	99.0%	0.1%	0.1%	98.7%	0.1%	0.1%	0.1%	
C	99.8%	0.2%	99.8%	0.4%	99.8%	0.2%	0.0%	0.0%	0.6%	0.5%	0.0%	0.0%	1.0%	0.0%	0.8%	0.0%	0.5%	0.0%	0.0%	0.7%	0.3%	0.0%	0.0%	
G	0.0%	0.2%	0.0%	0.3%	0.0%	2.9%	99.9%	99.8%	0.9%	0.2%	99.8%	99.8%	1.2%	99.9%	1.0%	99.9%	0.4%	99.9%	99.8%	0.5%	0.2%	99.8%	99.8%	
T	0.1%	0.1%	0.1%	0.1%	0.1%	0.0%	0.0%	0.1%	0.2%	99.1%	0.0%	0.0%	96.9%	0.0%	97.8%	0.0%	0.1%	0.0%	0.1%	0.2%	99.4%	0.0%	0.1%	

GFP Control	*JUP* Intron 10								*JUP* Exon 10												PAM			% Indel
AHCY Exon 9	G1	C2	G3	T4	G5	T6	A7	G8	G9	T10	G11	G12	A13	C14	C15	G16	G17	T18	A19	T20	C	G	G	0.00%
A	0.1%	0.0%	0.1%	0.0%	0.1%	0.0%	99.3%	0.2%	0.1%	0.2%	0.1%	0.1%	99.2%	0.1%	0.0%	0.1%	0.1%	0.0%	99.5%	0.0%	0.0%	0.1%	0.1%	
C	0.0%	99.8%	0.1%	0.3%	0.0%	0.2%	0.2%	0.2%	0.0%	0.4%	0.0%	0.0%	0.1%	99.7%	99.7%	0.0%	0.0%	0.2%	0.0%	0.2%	99.8%	0.0%	0.0%	
G	99.8%	0.1%	99.7%	0.2%	99.7%	0.2%	0.3%	99.3%	99.8%	0.4%	99.6%	99.8%	0.5%	0.1%	0.1%	99.7%	99.8%	0.2%	0.3%	0.2%	0.0%	99.7%	99.8%	
T	0.1%	0.1%	0.1%	99.5%	0.2%	99.6%	0.2%	0.2%	0.1%	99.0%	0.3%	0.1%	0.1%	0.1%	0.2%	0.1%	0.1%	99.6%	0.1%	99.5%	0.1%	0.1%	0.1%	

WT ABE																								% Indel
AHCY Exon 9	G1	C2	G3	T4	G5	T6	A7	G8	G9	T10	G11	G12	A13	C14	C15	G16	G17	T18	A19	T20	C	G	G	0.00%
A	0.1%	0.0%	0.1%	0.0%	0.1%	0.0%	75.4%	0.2%	0.1%	0.2%	0.1%	0.1%	99.2%	0.1%	0.0%	0.2%	0.1%	0.0%	99.6%	0.0%	0.0%	0.1%	0.1%	
C	0.0%	99.8%	0.0%	0.3%	0.0%	0.2%	0.2%	0.2%	0.0%	0.4%	0.0%	0.0%	0.1%	99.7%	99.7%	0.0%	0.0%	0.2%	0.0%	0.2%	99.9%	0.0%	0.0%	
G	99.8%	0.0%	99.8%	0.1%	99.7%	0.1%	24.1%	99.4%	99.8%	0.3%	99.7%	99.8%	0.5%	0.1%	0.1%	99.7%	99.8%	0.1%	0.3%	0.2%	0.0%	99.8%	99.8%	
T	0.1%	0.1%	0.1%	99.5%	0.2%	99.7%	0.3%	0.2%	0.1%	99.1%	0.2%	0.1%	0.1%	0.1%	0.2%	0.1%	0.1%	99.7%	0.1%	99.6%	0.1%	0.1%	0.1%	

ABE-UGI																								% Indel
AHCY Exon 9	G1	C2	G3	T4	G5	T6	A7	G8	G9	T10	G11	G12	A13	C14	C15	G16	G17	T18	A19	T20	C	G	G	0.00%
A	0.1%	0.0%	0.1%	0.0%	0.1%	0.0%	70.3%	0.2%	0.1%	0.2%	0.1%	0.1%	99.3%	0.1%	0.0%	0.2%	0.1%	0.0%	99.6%	0.0%	0.0%	0.1%	0.1%	
C	0.0%	99.8%	0.0%	0.3%	0.0%	0.2%	0.3%	0.2%	0.0%	0.4%	0.0%	0.0%	0.1%	99.7%	99.7%	0.0%	0.0%	0.2%	0.0%	0.2%	99.9%	0.0%	0.0%	
G	99.8%	0.1%	99.7%	0.2%	99.6%	0.1%	29.1%	99.3%	99.8%	0.4%	99.6%	99.8%	0.5%	0.1%	0.1%	99.7%	99.8%	0.2%	0.3%	0.2%	0.0%	99.8%	99.8%	
T	0.1%	0.1%	0.1%	99.5%	0.2%	99.6%	0.3%	0.3%	0.1%	99.0%	0.3%	0.1%	0.1%	0.1%	0.1%	0.1%	0.1%	99.6%	0.1%	99.5%	0.1%	0.1%	0.1%	

ABE-GGGGS5																								% Indel
AHCY Exon 9	G1	C2	G3	T4	G5	T6	A7	G8	G9	T10	G11	G12	A13	C14	C15	G16	G17	T18	A19	T20	C	G	G	0.00%
A	0.1%	0.0%	0.1%	0.0%	0.1%	0.0%	76.4%	0.3%	0.1%	0.2%	0.1%	0.1%	99.0%	0.1%	0.1%	0.2%	0.1%	0.0%	99.4%	0.1%	0.0%	0.1%	0.1%	
C	0.0%	99.7%	0.0%	0.2%	0.1%	0.2%	0.3%	0.2%	0.0%	0.4%	0.0%	0.0%	0.2%	99.7%	99.6%	0.0%	0.0%	0.2%	0.1%	0.2%	99.8%	0.0%	0.0%	
G	99.8%	0.1%	99.7%	0.2%	99.6%	0.2%	23.0%	99.1%	99.7%	0.5%	99.6%	99.7%	0.7%	0.1%	0.2%	99.7%	99.8%	0.3%	0.4%	0.3%	0.0%	99.7%	99.8%	
T	0.1%	0.2%	0.1%	99.5%	0.2%	99.6%	0.3%	0.4%	0.2%	98.9%	0.3%	0.2%	0.2%	0.1%	0.2%	0.1%	0.1%	99.6%	0.1%	99.4%	0.1%	0.1%	0.1%	

Supplementary Table S1: Raw base composition data and calculated % indels for HTS results for untreated HEK293T cells as well as cells transfected with WT ABE, ABE-UGI, and ABE-GGGGS5. Reported percentages are the mean values from two replicates.

CTNNA1 On-Target Site	Total Sequencing Reads with Target A Converted to G																						
On Target Score: 65.5	T1	C2	T3	G4	C5	A6	G7	A8	A9	A10	C11	A12	A13	A14	T15	C16	A17	T18	T19	G20	T	G	G
GFP						0.3%		0.2%	0.3%	0.3%		0.2%	0.2%	0.2%			0.2%						
WT ABE						6.5%		0.5%	0.5%	0.4%		0.3%	0.2%	0.2%			0.2%						

CTNNA1 Off-Target Site 1	Total Sequencing Reads with Target A Converted to G																						
Off Target Score: 2.7	T1	G2	T3	G4	A5	A6	G7	G8	A9	A10	C11	A12	A13	A14	T15	C16	A17	T18	T19	G20	A	G	G
GFP					0.3%	0.3%			0.3%	0.3%		0.2%	0.2%	0.2%			0.2%				0.3%		
WT ABE					0.3%	0.3%			0.2%	0.3%		0.2%	0.2%	0.2%			0.2%				0.2%		

CTNNA1 Off-Target Site 2	Total Sequencing Reads with Target A Converted to G																						
Off Target Score: 2.0	A1	C2	T3	G4	T5	A6	G7	A8	A9	A10	C11	A12	A13	A14	T15	C16	A17	T18	T19	T20	A	A	G
GFP	0.2%					0.2%		0.2%	0.3%	0.3%		0.2%	0.2%	0.2%			0.1%				0.1%	0.3%	
WT ABE	0.2%					0.2%		0.2%	0.2%	0.2%		0.2%	0.2%	0.2%			0.2%				0.1%	0.2%	

CTNNA1 Off-Target Site 3	Total Sequencing Reads with Target A Converted to G																						
Off Target Score: 0.8	T1	C2	T3	G4	T5	G6	G7	T8	A9	T10	C11	A12	A13	A14	T15	C16	A17	T18	T19	G20	A	G	G
GFP									0.2%			0.2%	0.1%	0.2%			0.1%				0.2%		
WT ABE									0.2%			0.2%	0.1%	0.2%			0.2%				0.2%		

CTNNA1 Off-Target Site 4	Total Sequencing Reads with Target A Converted to G																						
Off Target Score: 0.6	T1	C2	T3	G4	C5	A6	C7	A8	A9	A10	G11	A12	A13	A14	T15	C16	A17	T18	T19	C20	T	A	G
GFP						0.2%		0.2%	0.2%	0.1%		0.1%	0.1%	0.1%			0.1%					0.1%	
WT ABE						0.2%		0.2%	0.1%	0.1%		0.1%	0.3%	0.2%			0.1%					0.1%	

HSF1 On-Target Site	Total Sequencing Reads with Target A Converted to G																						
On Target Score: 44.1	T1	C2	C3	G4	C5	A6	G7	C8	T9	G10	T11	T12	C13	A14	G15	C16	C17	C18	C19	T20	C	G	G
GFP						1.4%								1.2%									
WT ABE						45.0%								1.2%									

HSF1 Off-Target Site 1	Total Sequencing Reads with Target A Converted to G																						
Off Target Score: 2.5	T1	C2	T3	C4	C5	A6	G7	C8	T9	C10	T11	T12	C13	A14	G15	C16	C17	C18	C19	T20	A	G	G
GFP						0.2%								0.2%							0.2%		
WT ABE						0.2%								0.2%							0.2%		

HSF1 Off-Target Site 2	Total Sequencing Reads with Target A Converted to G																						
Off Target Score: 1.7	A1	C2	C3	C4	C5	A6	G7	C8	T9	G10	C11	T12	C13	A14	G15	C16	C17	C18	C19	T20	G	G	G
GFP	1.2%					0.4%								0.2%									
WT ABE	0.8%					0.2%								0.3%									

HSF1 Off-Target Site 3	Total Sequencing Reads with Target A Converted to G																						
Off Target Score: 1.0	C1	C2	A3	G4	C5	A6	T7	G8	T9	G10	T11	T12	C13	A14	G15	C16	C17	C18	C19	T20	C	A	G
GFP			0.2%			0.2%								0.2%								0.2%	
WT ABE			0.2%			0.2%								0.2%								0.2%	

HSF1 Off-Target Site 4	Total Sequencing Reads with Target A Converted to G																						
Off Target Score: 0.8	T1	C2	T3	G4	A5	T6	G7	C8	T9	T10	T11	T12	C13	A14	G15	C16	C17	C18	C19	T20	T	G	G
GFP					0.3%									0.4%									
WT ABE					0.3%									0.4%									

JUP On-Target Site	Total Sequencing Reads with Target A Converted to G																						
On Target Score: 65.5	C1	A2	C3	A4	C5	A6	G7	G8	A9	T10	G11	G12	T13	G14	T15	G16	A17	G18	G19	A20	T	G	G
GFP		0.2%		0.3%		0.2%			0.3%								0.3%			0.3%			
WT ABE		0.3%		0.3%		2.2%			0.9%								0.4%			0.4%			

JUP Off-Target Site 1	Total Sequencing Reads with Target A Converted to G																						
Off Target Score: 38.7	C1	A2	C3	A4	C5	A6	G7	G8	A9	T10	G11	G12	A13	G14	T15	G16	A17	G18	G19	A20	T	G	G
GFP		0.2%		0.2%		0.2%			0.3%				0.3%				0.3%			0.2%			
WT ABE		0.2%		0.2%		0.5%			0.3%				0.3%				0.3%			0.2%			

JUP Off-Target Site 2	Total Sequencing Reads with Target A Converted to G																						
Off Target Score: 5.7	C1	A2	C3	A4	G5	A6	G7	A8	A9	T10	G11	G12	T13	G14	T15	G16	A17	G18	G19	A20	A	G	G
GFP		0.3%		0.2%		0.2%		0.2%	0.3%								0.2%			0.3%	0.3%		
WT ABE		0.3%		0.2%		0.2%		0.2%	0.3%								0.3%			0.2%	0.3%		

JUP Off-Target Site 3	Total Sequencing Reads with Target A Converted to G																						
Off Target Score: 1.4	G1	T2	C3	C4	C5	A6	G7	G8	A9	G10	G11	G12	T13	G14	T15	G16	A17	G18	G19	A20	C	A	G
GFP						0.1%			0.2%								99.9%			0.2%		0.2%	
WT ABE						0.2%			0.2%								0.2%			0.2%		0.3%	

JUP Off-Target Site 4	Total Sequencing Reads with Target A Converted to G																						
Off Target Score: 1.1	C1	C2	C3	A4	C5	G6	G7	G8	A9	T10	G11	G12	T13	G14	T15	G16	A17	G18	G19	C20	T	G	G
GFP				2.7%					0.8%								3.5%						
WT ABE				2.1%					0.6%								1.7%						

AHCY On-Target Site	Total Sequencing Reads with Target A Converted to G																						
On Target Score: 43.9	G1	C2	G3	T4	G5	T6	A7	G8	G9	T10	G11	G12	A13	C14	C15	G16	G17	T18	A19	T20	C	G	G
GFP							0.3%						0.6%						0.3%				
WT ABE							25.4%						0.5%						0.2%				

AHCY Off-Target Site 1	Total Sequencing Reads with Target A Converted to G																						
Off Target Score: 0.6	G1	A2	G3	T4	G5	T6	G7	G8	G9	T10	G11	G12	A13	C14	A15	G16	G17	T18	A19	T20	G	A	G
GFP		0.3%											0.4%		0.3%				0.2%			0.2%	
WT ABE		0.3%											0.3%		0.3%				0.1%			0.2%	

AHCY Off-Target Site 2	Total Sequencing Reads with Target A Converted to G																						
Off Target Score: 0.4	G1	A2	G3	T4	G5	T6	A7	T8	T9	T10	G11	G12	A13	C14	C15	G16	T17	T18	A19	T20	G	G	G
GFP		0.2%					0.1%						0.3%						0.1%				
WT ABE		0.2%					0.2%						0.3%						0.1%				

AHCY Off-Target Site 3	Total Sequencing Reads with Target A Converted to G																						
Off Target Score: 0.3	G1	C2	G3	G4	G5	T6	A7	G8	G9	A10	G11	A12	A13	C14	C15	G16	G17	T18	C19	T20	C	G	G
GFP							0.3%			0.4%		0.5%	1.2%										
WT ABE							0.3%			0.4%		0.4%	0.8%										

AHCY Off-Target Site 4	Total Sequencing Reads with Target A Converted to G																						
Off Target Score: 0.1	C1	G2	G3	T4	G5	T6	A7	G8	G9	T10	G11	G12	A13	G14	C15	T16	G17	T18	A19	T20	T	G	G
GFP							0.1%						0.3%						0.1%				
WT ABE							0.2%						0.3%						0.1%				

Supplementary Table S2: Base composition and %indels of predicted off-target sites [1] from HTS of genomic DNA from untreated HEK293T cells as well as cells transfected with plasmids encoding WT ABE and the corresponding sgRNA.

Target	Sequence (5' to 3')	PAM	On-Target Score	% Indels
CTNNA1 Exon 7	CTGCAGAAACAAATCATTG	TGG	65.5	8.49
HSF1 Exon 11	TCCGCAGCTGTTCAGCCCCT	TGG	44.1	16.48
JUP Exon 10	ACACAGGATGGTGTGAGGA	CGG	52.5	33.85
AHCY Exon 9	GCGTGTAGGTGGACCGGTAT	CGG	43.9	11.47

Supplementary Table S3: Predicted on-target activity for each sgRNA [2] and observed %indels from HTS of genomic DNA following transfection with plasmids encoding *wt* Cas9 and the corresponding sgRNA.

Designation	Target	Sequence (5' to 3')	PAM	On-Target Score	Off-Target Score
CTNNA1 Ex7 ABE S	CTNNA1 Exon 7	CACCGCTGCAGAAACAAATCATTG	TGG	65.5	50.7
CTNNA1 Ex7 ABE AS	CTNNA1 Exon 7	AAACCAATGATTTGTTTCTGCAGC	TGG	65.5	50.7
HSF1 Ex11 ABE S	HSF1 Exon 11	CACCGTCCGCAGCTGTTCAGCCCCT	CGG	44.1	63.7
HSF1 Ex11 ABE AS	HSF1 Exon 11	AAACAGGGGCTGAACAGCTGCGGAC	CGG	44.1	63.7
JUP Ex10 ABE S	JUP Exon 10	CACCGACACAGGATGGTGTGAGGA	TGG	52.5	28.1
JUP Ex10 ABE AS	JUP Exon 10	AAACTCCTCACACCATCCTGTGTC	TGG	52.5	28.1
AHCY Ex9 ABE S	AHCY Exon 9	CACCGGCGTGTAGGTGGACCGGTAT	CGG	43.9	49.0
AHCY Ex9 ABE AS	AHCY Exon 9	AAACATACCGGTCCACCTACACGCC	CGG	43.9	49.0
mCTNNB1 Ex11 S	Mouse CTNNB1 Exon 11	CACCGCTCCTAGGAGGGCGTGCGCA	TGG	40.4	83.7
mCTNNB1 Ex11 AS	Mouse CTNNB1 Exon 11	AAACTGCGCACGCCCTCCTAGGAGC	TGG	40.4	83.7
A-Rich Target 1 S	GAPDH Exon 1	CACCGTGAAAGAAAGAAAGGGGAGG	GGG	54.9	20.9
A-Rich Target 1 AS	GAPDH Exon 1	AAACCCTCCCCTTTCTTTCTTTCAC	GGG	54.9	20.9
A-Rich Target 2 S	GAPDH Intron 3	CACCGAATCTAGGAAAAGCATCACC	CGG	64.4	37.0
A-Rich Target 2 AS	GAPDH Intron 3	AAACGGTGATGCTTTTCCTAGATTC	CGG	64.4	37.0

Supplementary Table S4: Oligonucleotide sequences used to generate sgRNAs as well as predicted on-target scores[20] and off-target scores [1]. The splice acceptor adenine or the adenines within the A-rich target sgRNAs are highlighted in red.

Primer	Sequence (5' to 3')	WT Size (bp)	Skip Size (bp)
CTNNA1 Ex7 FW	CACCCTGATGTCGCAGCCTATA	484	280
CTNNA1 Ex7 REV	CTGAAACGTGGTCCATGACAGC	484	280
HSF1 Ex11 FW	TGCCTGGACAAGAATGAGCTCA	374	308
HSF1 Ex11REV	CTCTAGGAGACAGTGGGGTCCT	374	308
JUP Ex10 FW	TCTGTGCGTCTCAACTATGGCA	565	445
JUP Ex10 REV	GCTTCCGGTAGTCTGGGTTCTT	565	445
AHCY Ex9 FW	GTCAAGTGGCTCAACGAGAACG	441	246
AHCY Ex9 REV	TCCAAGACCACTGAGCTCATGG	441	246
mCTNNB1 Ex 11 FW	TGTGGTTAAACTCCTGCACCCA	371	251
mCTNNB1 Ex 11 REV	CCCCTGCAGCTACTCTTTGGAT	371	251
A-Rich Target 1 FW	ACACTGTCTCTCTCCCTAGGCA	N/A	N/A
A-Rich Target 1 REV	GCAGGACACTAGGGAGTCAAGG	N/A	N/A
A-Rich Target 2 FW	GCTTTCTTTCCTTTCGCGCTCT	N/A	N/A
A-Rich Target 2 REV	GTGGGAGATCTGGTTTCCGGAA	N/A	N/A

Supplementary Table S5: Nucleotide sequences of primers used for RT-PCR.

Primer	Sequence (5' to 3')
A-Rich Target 1 Seq	TCCCGAGCCTCCTTCCTCTC
A-Rich Target 2 Seq	ATCCCTGTCCGGATGCTG

Supplementary Table S6: Nucleotide sequences of primers used for Sanger sequencing of A-Rich Target PCR amplicons.

Primer	Sequence (5' to 3')	Template	PCR Cycles
CTNNA1 Ex7 ON FW	GTAGGCCATCTTCTGTGGGACA	gDNA	30
CTNNA1 Ex7 ON REV	TGTACTCCGAAAGCAGGTCCTG	gDNA	30
CTNNA1 Ex7 OFF1 FW	ATGTGCCCGATCTGCGATCTTA	gDNA	30
CTNNA1 Ex7 OFF1 REV	GCCAGTCTAACAGCATGCAGTG	gDNA	30
CTNNA1 Ex7 OFF2 FW	GCGAAAGGTGTGAACAGATGCT	gDNA	30
CTNNA1 Ex7 OFF2 REV	ACATATCCCGTGTTTGCTGCAC	gDNA	30
CTNNA1 Ex7 OFF3 FW	AGGAGACTGCACGTTCTTTGGA	gDNA	30
CTNNA1 Ex7 OFF3 REV	TTCCTCACCTCCAGGCTTCATG	gDNA	30
CTNNA1 Ex7 OFF4 FW	TTTCAATGCAAAGCTCCCCCAC	gDNA	30
CTNNA1 Ex7 OFF4 REV	TAAAGCCTGGCCTCGACATGAA	gDNA	30
CTNNA1 Ex7 cDNA FW	CACCCTGATGTCGCAGCCTATA	cDNA	30
CTNNA1 Ex7 cDNA REV	GCAAGTCCCTGGTCTTCTTGGT	cDNA	30
HSF1 Ex11 ON FW	CTGTTCTGACTTCCCTCCCTCC	gDNA	32
HSF1 Ex11 ON REV	TGGGACTTGGCTCACCTGAATC	gDNA	32
HSF1 Ex11 OFF1 FW	CTGTCAATAGGGCCTAGCACCA	gDNA	30
HSF1 Ex11 OFF1 REV	CTGCCAAGTGACCTCCTCTCAA	gDNA	30
HSF1 Ex11 OFF2 FW	CATCCACCACCAAGAGCTGAGA	gDNA	30
HSF1 Ex11 OFF2 REV	CCCACCCTCTCACTCTGTCTTG	gDNA	30
HSF1 Ex11 OFF3 FW	ACCACTCATTCTGGCATCGTGA	gDNA	30
HSF1 Ex11 OFF3 REV	CCTGCCACTCTCCACTTCTCTC	gDNA	30
HSF1 Ex11 OFF4 FW	TGTGCCGGATCTTAGCCTCAAA	gDNA	30
HSF1 Ex11 OFF4 REV	AAAGGAGGAGAGCTGCGTTCAT	gDNA	30
HSF1 Ex11 cDNA FW	TGCCTGGACAAGAATGAGCTCA	cDNA	30
HSF1 Ex11 cDNA REV	TCGGAGAAGTAGGAGCCCTCTC	cDNA	30
JUP Ex10 ON FW	CTGTGGGTGTGTGTGTGAATGG	gDNA	30
JUP Ex10 ON REV	GCAGGGGGTTGCTAAGTAGTCA	gDNA	30
JUP Ex10 OFF1 FW	TGCCTCCTGCTTGTACTCTTCC	gDNA	30
JUP Ex10 OFF1 REV	GCTTACTGGGCCATCTCAGTGA	gDNA	30
JUP Ex10 OFF2 FW	GTAGGGTTTGGCCTTTTGCTCC	gDNA	30
JUP Ex10 OFF2 REV	CCCCAGGTAAAAGCACCAGGTA	gDNA	30
JUP Ex10 OFF3 FW	TGTCTGTCCTGGTCACGGATTC	gDNA	30
JUP Ex10 OFF3 REV	CCTGTGGTTCTGGGAGTCTCTG	gDNA	30
JUP Ex10 OFF4 FW	AAAGGGACTGTGGCATCTCCTC	gDNA	30
JUP Ex10 OFF4 REV	TCACAGGCATCAAGGTGGTAGG	gDNA	30
JUP Ex10 cDNA FW	TCTGTGCGTCTCAACTATGGCA	cDNA	30
JUP Ex10 cDNA REV	TGTTCTCCACCGACGAGTACAG	cDNA	30
AHCY Ex9 gON FW	GAGACGGGCTTTCACTGTGTTG	gDNA	30
AHCY Ex9 gON REV	AACGGGGTACTTGTCTGGATGG	gDNA	30
AHCY Ex9 OFF1 FW	TGCTTTTGAACATGCCAGCCAT	gDNA	30
AHCY Ex9 OFF1 REV	CCAGGAAGGCTTTGCTTCCAAG	gDNA	30
AHCY Ex9 OFF2 FW	AACCCCTGAACGAGTGGGAATT	gDNA	30
AHCY Ex9 OFF2 REV	TCCCACAAATCCTCCACTGGTG	gDNA	30
AHCY Ex9 OFF3 FW	ATCCGGTTCAGTGGACTCTGTG	gDNA	30
AHCY Ex9 OFF3 REV	AATGTCTGCGGGTCTCTGTCTC	gDNA	30
AHCY Ex9 OFF4 FW	GGAACACAGGGTTGATGCCATG	gDNA	30
AHCY Ex9 OFF4 REV	TCCTGAAGTGCGAGTACTGTGG	gDNA	30
AHCY Ex9 cDNA FW	CATCTTTGTCACCACCACAGGC	cDNA	30
AHCY Ex9 cDNA REV	AGGTACTGGGCTTGCTTCTCAG	cDNA	30

Supplementary Table S7: Nucleotide sequences of PCR primers used to generate amplicons for HTS. The number of cycles and type of template DNA used in the PCR is indicated.

Supplementary Sequences 1: Amino acid sequences of the ABEs used in this study.

Orange – Adenine Deaminase Domain

Green – Linker Region

Black – Cas9-D10A

Purple – UGI Domain

Red – N-terminal Intein

Blue – C-terminal Intein

Magenta – NLS

Dark Green – 3x HA Tag

wt ABE

MSEVEFSHEYWMRHALTLAKRAWDEREVPVGAVLVHNNRVIGEGWNRPIGRHDPTAHAEIMA
LRQGGLVMQNYRLIDATLYVTLEPCVMCAGAMIHSRIGRVVFGARDAKTGAAGSLMDVLHHPG
MNHRVEITEGILADECAALLSDFFRMRRQEIKAQKKAQSSTDSGGSSGGSSGSETPGTSESAT
PESSGGSSGGSSEVEFSHEYWMRHALTLAKRARDEREVPVGAVLVLNNRVIGEGWNRAIGLH
DPTAHAEIMALRQGGLVMQNYRLIDATLYVTFEPCVMCAGAMIHSRIGRVVFGVRNAKTGAAG
SLMDVLHYPGMNHRVEITEGILADECAALLCYFFRMPRQVFNAQKKAQSSTDSGGSSGGSSG
SETPGTSESATPESSGGSSGGSDKKYSIGLAIGTNSVGWAVITDEYKVPSKKFKVLGNTDRHSI
KKNLIGALLFDSGETAEATRLKRTARRRYTRRKNRICYLQEIFSNEMAKVDDSFFHRLEESFLVE
EDKKHERHPIFGNIVDEVAYHEKYPTIYHLRKKLVDSTDKADLRLIYLALAHMIKFRGHFLIEGDL
NPDNSDVDKLFIQLVQTYNQLFEENPINASGVDAKAILSARLSKSRRLENLIAQLPGEKKNGLFG
NLIALSLGLTPNFKSNFDLAEDAKLQLSKDTYDDDLDNLLAQIGDQYADLFLAAKNLSDAILLSDIL
RVNTEITKAPLSASMIKRYDEHHQDLTLLKALVRQQLPEKYKEIFFDQSKNGYAGYIDGGASQE
EFYKFIKPILEKMDGTEELLVKLNREDLLRKQRTFDNGSIPHQIHLGELHAILRRQEDFYPFLKDN
REKIEKILTFRIPYYVGPLARGNSRFAWMTRKSEETITPWNFEEVVDKGASAQSFIERMTNFDK
NLPNEKVLPKHSLLYEYFTVYNELTKVKYVTEGMRKPAFLSGEQKKAIVDLLFKTNRKVTVKQL
KEDYFKKIECFDSVEISGVEDRFNASLGTYHDLLKIIKDKDFLDNEENEDILEDIVLTLTLFEDREM
IEERLKTYAHLFDDKVMKQLKRRRYTGWGRLSRKLINGIRDKQSGKTILDFLKSDGFANRNFMQ
LIHDDSLTFKEDIQKAQVSGQGDSLHEHIANLAGSPAIKKGILQTVKVVDELVKVMGRHKPENIVI
EMARENQTTQKGQKNSRERMKRIEEGIKELGSQILKEHPVENTQLQNEKLYLYYLQNGRDMYV
DQELDINRLSDYDVDHIVPQSFLKDDSIDNKVLTRSDKNRGKSDNVPSEEVVKKMKNYWRQLL
NAKLITQRKFDNLTKAERGGLSELDKAGFIKRQLVETRQITKHVAQILDSRMNTKYDENDKLIRE
VKVITLKSKLVSDFRKDFQFYKVREINNYHHAHDAYLNAVVGTALIKKYPKLESEFVYGDYKVYD
VRKMIAKSEQEIGKATAKYFFYSNIMNFFKTEITLANGEIRKRPLIETNGETGEIVWDKGRDFATV
RKVLSMPQVNIVKKTEVQTGGFSKESILPKRNSDKLIARKKDWDPKKYGGFDSPTVAYSVLVVA
KVEKGKSKKLKSVKELLGITIMERSSFEKNPIDFLEAKGYKEVKKDLIIKLPKYSLFELENGRKRM
LASAGELQKGNELALPSKYVNFLYLASHYEKLKGSPEDNEQKQLFVEQHKHYLDEIIEQISEFSK
RVILADANLDKVLSAYNKHRDKPIREQAENIIHLFTLTNLGAPAAFKYFDTTIDRKRYTSTKEVLD
ATLIHQSITGLYETRIDLSQLGGDSRADPKKKRKV*

ABE-AP_5

MSEVEFSHEYWMRHALTLAKRAWDEREVPVGAVLVHNNRVIGEGWNRPIGRHDPTAHAEIMA
LRQGGLVMQNYRLIDATLYVTLEPCVMCAGAMIHSRIGRVVFGARDAKTGAAGSLMDVLHHPG
MNHRVEITEGILADECAALLSDFFRMRRQEIKAQKKAQSSTDSGGSSGGSSGSETPGTSESAT

PESSGGSSGGSSEVEFSHEYWMRHALTLAKRARDEREVPVGAVLVLNNRVIGEGWNRAIGLH
DPTAHAEIMALRQGGLVMQNYRLIDATLYVTFEPCVMCAGAMIHSRIGRVVFGVRNAKTGAAG
SLMDVLHYPGMNHRVEITEGILADECAALLCYFFRMPRQVFNAQKKAQSSTDASAPAPAPAPA
PGTDKKYSIGLAIGTNSVGWAVITDEYKVPSKKFKVLGNTDRHSIKKNLIGALLFDSGETAEATR
LKRTARRRYTRRKNRICYLQEIFSNEMAKVDDSFFHRLEESFLVEEDKKHERHPIFGNIVDEVAY
HEKYPTIYHLRKKLVDSTDKADLRLIYLALAHMIKFRGHFLIEGDLNPDNSDVDKLFIQLVQTYNQ
LFEENPINASGVDAKAILSARLSKSRRLENLIAQLPGEKKNGLFGNLIALSLGLTPNFKSNFDLAE
DAKLQLSKDTYDDDLDNLLAQIGDQYADLFLAAKNLSDAILLSDILRVNTEITKAPLSASMIKRYD
EHHQDLTLLKALVRQQLPEKYKEIFFDQSKNGYAGYIDGGASQEEFYKFIKPILEKMDGTEELLV
KLNREDLLRKQRTFDNGSIPHQIHLGELHAILRRQEDFYPFLKDNREKIEKILTFRIPYYVGPLAR
GNSRFAWMTRKSEETITPWNFEEVVDKGASAQSFIERMTNFDKNLPNEKVLPKHSLLYEYFTV
YNELTKVKYVTEGMRKPAFLSGEQKKAIVDLLFKTNRKVTVKQLKEDYFKKIECFDSVEISGVED
RFNASLGTYHDLLKIIKDKDFLDNEENEDILEDIVLTLTLFEDREMIEERLKTYAHLFDDKVMKQL
KRRRYTGWGRLSRKLINGIRDKQSGKTILDFLKSDGFANRNFMQLIHDDSLTFKEDIQKAQVSG
QGDSLHEHIANLAGSPAIKKGILQTVKVVDELVKVMGRHKPENIVIEMARENQTTQKGQKNSRE
RMKRIEEGIKELGSQILKEHPVENTQLQNEKLYLYYLQNGRDMYVDQELDINRLSDYDVDHIVP
QSFLKDDSIDNKVLTRSDKNRGKSDNVPSEEVVKKMKNYWRQLLNAKLITQRKFDNLTKAERG
GLSELDKAGFIKRQLVETRQITKHVAQILDSRMNTKYDENDKLIREVKVITLKSKLVSDFRKDFQF
YKVREINNYHHAHDAYLNAVVGTALIKKYPKLESEFVYGDYKVYDVRKMIAKSEQEIGKATAKYF
FYSNIMNFFKTEITLANGEIRKRPLIETNGETGEIVWDKGRDFATVRKVLSMPQVNIVKKTEVQT
GGFSKESILPKRNSDKLIARKKDWDPKKYGGFDSPTVAYSVLVVAKVEKGKSKKLKSVKELLGI
TIMERSSFEKNPIDFLEAKGYKEVKKDLIIKLPKYSLFELENGRKRMLASAGELQKGNELALPSK
YVNFLYLASHYEKLKGSPEDNEQKQLFVEQHKHYLDEIIEQISEFSKRVILADANLDKVLSAYNK
HRDKPIREQAENIIHLFTLTNLGAPAAFKYFDTTIDRKRYTSTKEVLDATLIHQSITGLYETRIDLS
QLGGDSRADPKKKRKV*

ABE-GGGGS$_5$

MSEVEFSHEYWMRHALTLAKRAWDEREVPVGAVLVHNNRVIGEGWNRPIGRHDPTAHAEIMA
LRQGGLVMQNYRLIDATLYVTLEPCVMCAGAMIHSRIGRVVFGARDAKTGAAGSLMDVLHHPG
MNHRVEITEGILADECAALLSDFFRMRRQEIKAQKKAQSSTDSGGSSGGSSGSETPGTSESAT
PESSGGSSGGSSEVEFSHEYWMRHALTLAKRARDEREVPVGAVLVLNNRVIGEGWNRAIGLH
DPTAHAEIMALRQGGLVMQNYRLIDATLYVTFEPCVMCAGAMIHSRIGRVVFGVRNAKTGAAG
SLMDVLHYPGMNHRVEITEGILADECAALLCYFFRMPRQVFNAQKKAQSSTDASGGGGSGGG
GSGGGGSGGGGSGGGGSGTDKKYSIGLAIGTNSVGWAVITDEYKVPSKKFKVLGNTDRHSIK
KNLIGALLFDSGETAEATRLKRTARRRYTRRKNRICYLQEIFSNEMAKVDDSFFHRLEESFLVEE
DKKHERHPIFGNIVDEVAYHEKYPTIYHLRKKLVDSTDKADLRLIYLALAHMIKFRGHFLIEGDLN
PDNSDVDKLFIQLVQTYNQLFEENPINASGVDAKAILSARLSKSRRLENLIAQLPGEKKNGLFGN
LIALSLGLTPNFKSNFDLAEDAKLQLSKDTYDDDLDNLLAQIGDQYADLFLAAKNLSDAILLSDILR
VNTEITKAPLSASMIKRYDEHHQDLTLLKALVRQQLPEKYKEIFFDQSKNGYAGYIDGGASQEEF
YKFIKPILEKMDGTEELLVKLNREDLLRKQRTFDNGSIPHQIHLGELHAILRRQEDFYPFLKDNRE
KIEKILTFRIPYYVGPLARGNSRFAWMTRKSEETITPWNFEEVVDKGASAQSFIERMTNFDKNLP
NEKVLPKHSLLYEYFTVYNELTKVKYVTEGMRKPAFLSGEQKKAIVDLLFKTNRKVTVKQLKED
YFKKIECFDSVEISGVEDRFNASLGTYHDLLKIIKDKDFLDNEENEDILEDIVLTLTLFEDREMIEE
RLKTYAHLFDDKVMKQLKRRRYTGWGRLSRKLINGIRDKQSGKTILDFLKSDGFANRNFMQLIH
DDSLTFKEDIQKAQVSGQGDSLHEHIANLAGSPAIKKGILQTVKVVDELVKVMGRHKPENIVIEM
ARENQTTQKGQKNSRERMKRIEEGIKELGSQILKEHPVENTQLQNEKLYLYYLQNGRDMYVDQ
ELDINRLSDYDVDHIVPQSFLKDDSIDNKVLTRSDKNRGKSDNVPSEEVVKKMKNYWRQLLNA
KLITQRKFDNLTKAERGGLSELDKAGFIKRQLVETRQITKHVAQILDSRMNTKYDENDKLIREVK
VITLKSKLVSDFRKDFQFYKVREINNYHHAHDAYLNAVVGTALIKKYPKLESEFVYGDYKVYDVR

KMIAKSEQEIGKATAKYFFYSNIMNFFKTEITLANGEIRKRPLIETNGETGEIVWDKGRDFATVRK
VLSMPQVNIVKKTEVQTGGFSKESILPKRNSDKLIARKKDWDPKKYGGFDSPTVAYSVLVVAKV
EKGKSKKLKSVKELLGITIMERSSFEKNPIDFLEAKGYKEVKKDLIIKLPKYSLFELENGRKRMLA
SAGELQKGNELALPSKYVNFLYLASHYEKLKGSPEDNEQKQLFVEQHKHYLDEIIEQISEFSKRV
ILADANLDKVLSAYNKHRDKPIREQAENIIHLFTLTNLGAPAAFKYFDTTIDRKRYTSTKEVLDATL
IHQSITGLYETRIDLSQLGGDSRADPKKKRKV*

ABE-Dual

MSEVEFSHEYWMRHALTLAKRAWDEREVPVGAVLVHNNRVIGEGWNRPIGRHDPTAHAEIMA
LRQGGLVMQNYRLIDATLYVTLEPCVMCAGAMIHSRIGRVVFGARDAKTGAAGSLMDVLHHPG
MNHRVEITEGILADECAALLSDFFRMRRQEIKAQKKAQSSTDSGGSSGGSSGSETPGTSESAT
PESSGGSSGGSSEVEFSHEYWMRHALTLAKRARDEREVPVGAVLVLNNRVIGEGWNRAIGLH
DPTAHAEIMALRQGGLVMQNYRLIDATLYVTFEPCVMCAGAMIHSRIGRVVFGVRNAKTGAAG
SLMDVLHYPGMNHRVEITEGILADECAALLCYFFRMPRQVFNAQKKAQSSTDASSGGSSGGS
SGSETPGTSESATPESSGGSSGGSGGGGSGGGGSGGGGSGGGGSGGGGSGTDKKYSIGLA
IGTNSVGWAVITDEYKVPSKKFKVLGNTDRHSIKKNLIGALLFDSGETAEATRLKRTARRRYTRR
KNRICYLQEIFSNEMAKVDDSFFHRLEESFLVEEDKKHERHPIFGNIVDEVAYHEKYPTIYHLRK
KLVDSTDKADLRLIYLALAHMIKFRGHFLIEGDLNPDNSDVDKLFIQLVQTYNQLFEENPINASGV
DAKAILSARLSKSRRLENLIAQLPGEKKNGLFGNLIALSLGLTPNFKSNFDLAEDAKLQLSKDTYD
DDLDNLLAQIGDQYADLFLAAKNLSDAILLSDILRVNTEITKAPLSASMIKRYDEHHQDLTLLKALV
RQQLPEKYKEIFFDQSKNGYAGYIDGGASQEEFYKFIKPILEKMDGTEELLVKLNREDLLRKQRT
FDNGSIPHQIHLGELHAILRRQEDFYPFLKDNREKIEKILTFRIPYYVGPLARGNSRFAWMTRKS
EETITPWNFEEVVDKGASAQSFIERMTNFDKNLPNEKVLPKHSLLYEYFTVYNELTKVKYVTEG
MRKPAFLSGEQKKAIVDLLFKTNRKVTVKQLKEDYFKKIECFDSVEISGVEDRFNASLGTYHDLL
KIIKDKDFLDNEENEDILEDIVLTLTLFEDREMIEERLKTYAHLFDDKVMKQLKRRRYTGWGRLS
RKLINGIRDKQSGKTILDFLKSDGFANRNFMQLIHDDSLTFKEDIQKAQVSGQGDSLHEHIANLA
GSPAIKKGILQTVKVVDELVKVMGRHKPENIVIEMARENQTTQKGQKNSRERMKRIEEGIKELG
SQILKEHPVENTQLQNEKLYLYYLQNGRDMYVDQELDINRLSDYDVDHIVPQSFLKDDSIDNKV
LTRSDKNRGKSDNVPSEEVVKKMKNYWRQLLNAKLITQRKFDNLTKAERGGLSELDKAGFIKR
QLVETRQITKHVAQILDSRMNTKYDENDKLIREVKVITLKSKLVSDFRKDFQFYKVREINNYHHA
HDAYLNAVVGTALIKKYPKLESEFVYGDYKVYDVRKMIAKSEQEIGKATAKYFFYSNIMNFFKTEI
TLANGEIRKRPLIETNGETGEIVWDKGRDFATVRKVLSMPQVNIVKKTEVQTGGFSKESILPKRN
SDKLIARKKDWDPKKYGGFDSPTVAYSVLVVAKVEKGKSKKLKSVKELLGITIMERSSFEKNPID
FLEAKGYKEVKKDLIIKLPKYSLFELENGRKRMLASAGELQKGNELALPSKYVNFLYLASHYEKL
KGSPEDNEQKQLFVEQHKHYLDEIIEQISEFSKRVILADANLDKVLSAYNKHRDKPIREQAENIIH
LFTLTNLGAPAAFKYFDTTIDRKRYTSTKEVLDATLIHQSITGLYETRIDLSQLGGDSRADPKKKR
KV*

ABE-EAAA$_5$

MSEVEFSHEYWMRHALTLAKRAWDEREVPVGAVLVHNNRVIGEGWNRPIGRHDPTAHAEIMA
LRQGGLVMQNYRLIDATLYVTLEPCVMCAGAMIHSRIGRVVFGARDAKTGAAGSLMDVLHHPG
MNHRVEITEGILADECAALLSDFFRMRRQEIKAQKKAQSSTDSGGSSGGSSGSETPGTSESAT
PESSGGSSGGSSEVEFSHEYWMRHALTLAKRARDEREVPVGAVLVLNNRVIGEGWNRAIGLH
DPTAHAEIMALRQGGLVMQNYRLIDATLYVTFEPCVMCAGAMIHSRIGRVVFGVRNAKTGAAG
SLMDVLHYPGMNHRVEITEGILADECAALLCYFFRMPRQVFNAQKKAQSSTDASEAAAEAAAE
AAAEAAAEAAAGTDKKYSIGLAIGTNSVGWAVITDEYKVPSKKFKVLGNTDRHSIKKNLIGALLF
DSGETAEATRLKRTARRRYTRRKNRICYLQEIFSNEMAKVDDSFFHRLEESFLVEEDKKHERHP
IFGNIVDEVAYHEKYPTIYHLRKKLVDSTDKADLRLIYLALAHMIKFRGHFLIEGDLNPDNSDVDK

LFIQLVQTYNQLFEENPINASGVDAKAILSARLSKSRRLENLIAQLPGEKKNGLFGNLIALSLGLT
PNFKSNFDLAEDAKLQLSKDTYDDDLDNLLAQIGDQYADLFLAAKNLSDAILLSDILRVNTEITKA
PLSASMIKRYDEHHQDLTLLKALVRQQLPEKYKEIFFDQSKNGYAGYIDGGASQEEFYKFIKPIL
EKMDGTEELLVKLNREDLLRKQRTFDNGSIPHQIHLGELHAILRRQEDFYPFLKDNREKIEKILTF
RIPYYVGPLARGNSRFAWMTRKSEETITPWNFEEVVDKGASAQSFIERMTNFDKNLPNEKVLP
KHSLLYEYFTVYNELTKVKYVTEGMRKPAFLSGEQKKAIVDLLFKTNRKVTVKQLKEDYFKKIEC
FDSVEISGVEDRFNASLGTYHDLLKIIKDKDFLDNEENEDILEDIVLTLTLFEDREMIEERLKTYAH
LFDDKVMKQLKRRRYTGWGRLSRKLINGIRDKQSGKTILDFLKSDGFANRNFMQLIHDDSLTFK
EDIQKAQVSGQGDSLHEHIANLAGSPAIKKGILQTVKVVDELVKVMGRHKPENIVIEMARENQTT
QKGQKNSRERMKRIEEGIKELGSQILKEHPVENTQLQNEKLYLYYLQNGRDMYVDQELDINRLS
DYDVDHIVPQSFLKDDSIDNKVLTRSDKNRGKSDNVPSEEVVKKMKNYWRQLLNAKLITQRKF
DNLTKAERGGLSELDKAGFIKRQLVETRQITKHVAQILDSRMNTKYDENDKLIREVKVITLKSKLV
SDFRKDFQFYKVREINNYHHAHDAYLNAVVGTALIKKYPKLESEFVYGDYKVYDVRKMIAKSEQ
EIGKATAKYFFYSNIMNFFKTEITLANGEIRKRPLIETNGETGEIVWDKGRDFATVRKVLSMPQV
NIVKKTEVQTGGFSKESILPKRNSDKLIARKKDWDPKKYGGFDSPTVAYSVLVVAKVEKGKSKK
LKSVKELLGITIMERSSFEKNPIDFLEAKGYKEVKKDLIIKLPKYSLFELENGRKRMLASAGELQK
GNELALPSKYVNFLYLASHYEKLKGSPEDNEQKQLFVEQHKHYLDEIIEQISEFSKRVILADANL
DKVLSAYNKHRDKPIREQAENIIHLFTLTNLGAPAAFKYFDTTIDRKRYTSTKEVLDATLIHQSITG
LYETRIDLSQLGGDSRADPKKKRKV*

ABE-UGI

MSEVEFSHEYWMRHALTLAKRAWDEREVPVGAVLVHNNRVIGEGWNRPIGRHDPTAHAEIMA
LRQGGLVMQNYRLIDATLYVTLEPCVMCAGAMIHSRIGRVVFGARDAKTGAAGSLMDVLHHPG
MNHRVEITEGILADECAALLSDFFRMRRQEIKAQKKAQSSTDSGGSSGGSSGSETPGTSESAT
PESSGGSSGGSSEVEFSHEYWMRHALTLAKRARDEREVPVGAVLVLNNRVIGEGWNRAIGLH
DPTAHAEIMALRQGGLVMQNYRLIDATLYVTFEPCVMCAGAMIHSRIGRVVFGVRNAKTGAAG
SLMDVLHYPGMNHRVEITEGILADECAALLCYFFRMPRQVFNAQKKAQSSTDSGGSSGGSSG
SETPGTSESATPESSGGSSGGSDKKYSIGLAIGTNSVGWAVITDEYKVPSKKFKVLGNTDRHSI
KKNLIGALLFDSGETAEATRLKRTARRRYTRRKNRICYLQEIFSNEMAKVDDSFFHRLEESFLVE
EDKKHERHPIFGNIVDEVAYHEKYPTIYHLRKKLVDSTDKADLRLIYLALAHMIKFRGHFLIEGDL
NPDNSDVDKLFIQLVQTYNQLFEENPINASGVDAKAILSARLSKSRRLENLIAQLPGEKKNGLFG
NLIALSLGLTPNFKSNFDLAEDAKLQLSKDTYDDDLDNLLAQIGDQYADLFLAAKNLSDAILLSDIL
RVNTEITKAPLSASMIKRYDEHHQDLTLLKALVRQQLPEKYKEIFFDQSKNGYAGYIDGGASQE
EFYKFIKPILEKMDGTEELLVKLNREDLLRKQRTFDNGSIPHQIHLGELHAILRRQEDFYPFLKDN
REKIEKILTFRIPYYVGPLARGNSRFAWMTRKSEETITPWNFEEVVDKGASAQSFIERMTNFDK
NLPNEKVLPKHSLLYEYFTVYNELTKVKYVTEGMRKPAFLSGEQKKAIVDLLFKTNRKVTVKQL
KEDYFKKIECFDSVEISGVEDRFNASLGTYHDLLKIIKDKDFLDNEENEDILEDIVLTLTLFEDREM
IEERLKTYAHLFDDKVMKQLKRRRYTGWGRLSRKLINGIRDKQSGKTILDFLKSDGFANRNFMQ
LIHDDSLTFKEDIQKAQVSGQGDSLHEHIANLAGSPAIKKGILQTVKVVDELVKVMGRHKPENIVI
EMARENQTTQKGQKNSRERMKRIEEGIKELGSQILKEHPVENTQLQNEKLYLYYLQNGRDMYV
DQELDINRLSDYDVDHIVPQSFLKDDSIDNKVLTRSDKNRGKSDNVPSEEVVKKMKNYWRQLL
NAKLITQRKFDNLTKAERGGLSELDKAGFIKRQLVETRQITKHVAQILDSRMNTKYDENDKLIRE
VKVITLKSKLVSDFRKDFQFYKVREINNYHHAHDAYLNAVVGTALIKKYPKLESEFVYGDYKVYD
VRKMIAKSEQEIGKATAKYFFYSNIMNFFKTEITLANGEIRKRPLIETNGETGEIVWDKGRDFATV
RKVLSMPQVNIVKKTEVQTGGFSKESILPKRNSDKLIARKKDWDPKKYGGFDSPTVAYSVLVVA
KVEKGKSKKLKSVKELLGITIMERSSFEKNPIDFLEAKGYKEVKKDLIIKLPKYSLFELENGRKRM
LASAGELQKGNELALPSKYVNFLYLASHYEKLKGSPEDNEQKQLFVEQHKHYLDEIIEQISEFSK
RVILADANLDKVLSAYNKHRDKPIREQAENIIHLFTLTNLGAPAAFKYFDTTIDRKRYTSTKEVLD
ATLIHQSITGLYETRIDLSQLGGDSRADSGGSTNLSDIIEKETGKQLVIQESILMLPEEVEEVIGNK
PESDILVHTAYDESTDENVMLLTSDAPEYKPWALVIQDSNGENKIKMLSGGSPKKKRKV*

ABE-GGGGS$_1$-UGI

MSEVEFSHEYWMRHALTLAKRAWDEREVPVGAVLVHNNRVIGEGWNRPIGRHDPTAHAEIMA
LRQGGLVMQNYRLIDATLYVTLEPCVMCAGAMIHSRIGRVVFGARDAKTGAAGSLMDVLHHPG
MNHRVEITEGILADECAALLSDFFRMRRQEIKAQKKAQSSTDSGGSSGGSSGSETPGTSESAT
PESSGGSSGGSSEVEFSHEYWMRHALTLAKRARDEREVPVGAVLVLNNRVIGEGWNRAIGLH
DPTAHAEIMALRQGGLVMQNYRLIDATLYVTFEPCVMCAGAMIHSRIGRVVFGVRNAKTGAAG
SLMDVLHYPGMNHRVEITEGILADECAALLCYFFRMPRQVFNAQKKAQSSTDASGGGGSGTD
KKYSIGLAIGTNSVGWAVITDEYKVPSKKFKVLGNTDRHSIKKNLIGALLFDSGETAEATRLKRTA
RRRYTRRKNRICYLQEIFSNEMAKVDDSFFHRLEESFLVEEDKKHERHPIFGNIVDEVAYHEKY
PTIYHLRKKLVDSTDKADLRLIYLALAHMIKFRGHFLIEGDLNPDNSDVDKLFIQLVQTYNQLFEE
NPINASGVDAKAILSARLSKSRRLENLIAQLPGEKKNGLFGNLIALSLGLTPNFKSNFDLAEDAKL
QLSKDTYDDDLDNLLAQIGDQYADLFLAAKNLSDAILLSDILRVNTEITKAPLSASMIKRYDEHHQ
DLTLLKALVRQQLPEKYKEIFFDQSKNGYAGYIDGGASQEEFYKFIKPILEKMDGTEELLVKLNR
EDLLRKQRTFDNGSIPHQIHLGELHAILRRQEDFYPFLKDNREKIEKILTFRIPYYVGPLARGNSR
FAWMTRKSEETITPWNFEEVVDKGASAQSFIERMTNFDKNLPNEKVLPKHSLLYEYFTVYNELT
KVKYVTEGMRKPAFLSGEQKKAIVDLLFKTNRKVTVKQLKEDYFKKIECFDSVEISGVEDRFNA
SLGTYHDLLKIIKDKDFLDNEENEDILEDIVLTLTLFEDREMIEERLKTYAHLFDDKVMKQLKRRR
YTGWGRLSRKLINGIRDKQSGKTILDFLKSDGFANRNFMQLIHDDSLTFKEDIQKAQVSGQGDS
LHEHIANLAGSPAIKKGILQTVKVVDELVKVMGRHKPENIVIEMARENQTTQKGQKNSRERMKRI
EEGIKELGSQILKEHPVENTQLQNEKLYLYYLQNGRDMYVDQELDINRLSDYDVDHIVPQSFLK
DDSIDNKVLTRSDKNRGKSDNVPSEEVVKKMKNYWRQLLNAKLITQRKFDNLTKAERGGLSEL
DKAGFIKRQLVETRQITKHVAQILDSRMNTKYDENDKLIREVKVITLKSKLVSDFRKDFQFYKVR
EINNYHHAHDAYLNAVVGTALIKKYPKLESEFVYGDYKVYDVRKMIAKSEQEIGKATAKYFFYSN
IMNFFKTEITLANGEIRKRPLIETNGETGEIVWDKGRDFATVRKVLSMPQVNIVKKTEVQTGGFS
KESILPKRNSDKLIARKKDWDPKKYGGFDSPTVAYSVLVVAKVEKGKSKKLKSVKELLGITIMER
SSFEKNPIDFLEAKGYKEVKKDLIIKLPKYSLFELENGRKRMLASAGELQKGNELALPSKYVNFL
YLASHYEKLKGSPEDNEQKQLFVEQHKHYLDEIIEQISEFSKRVILADANLDKVLSAYNKHRDKPI
REQAENIIHLFTLTNLGAPAAFKYFDTTIDRKRYTSTKEVLDATLIHQSITGLYETRIDLSQLGGDS
RADSGGSTNLSDIIEKETGKQLVIQESILMLPEEVEEVIGNKPESDILVHTAYDESTDENVMLLTS
DAPEYKPWALVIQDSNGENKIKMLSGGSPKKKRKV*

ABE-GGGGS$_2$-UGI

MSEVEFSHEYWMRHALTLAKRAWDEREVPVGAVLVHNNRVIGEGWNRPIGRHDPTAHAEIMA
LRQGGLVMQNYRLIDATLYVTLEPCVMCAGAMIHSRIGRVVFGARDAKTGAAGSLMDVLHHPG
MNHRVEITEGILADECAALLSDFFRMRRQEIKAQKKAQSSTDSGGSSGGSSGSETPGTSESAT
PESSGGSSGGSSEVEFSHEYWMRHALTLAKRARDEREVPVGAVLVLNNRVIGEGWNRAIGLH
DPTAHAEIMALRQGGLVMQNYRLIDATLYVTFEPCVMCAGAMIHSRIGRVVFGVRNAKTGAAG
SLMDVLHYPGMNHRVEITEGILADECAALLCYFFRMPRQVFNAQKKAQSSTDASGGGGSGGG
GSGTDKKYSIGLAIGTNSVGWAVITDEYKVPSKKFKVLGNTDRHSIKKNLIGALLFDSGETAEAT
RLKRTARRRYTRRKNRICYLQEIFSNEMAKVDDSFFHRLEESFLVEEDKKHERHPIFGNIVDEVA
YHEKYPTIYHLRKKLVDSTDKADLRLIYLALAHMIKFRGHFLIEGDLNPDNSDVDKLFIQLVQTYN
QLFEENPINASGVDAKAILSARLSKSRRLENLIAQLPGEKKNGLFGNLIALSLGLTPNFKSNFDLA
EDAKLQLSKDTYDDDLDNLLAQIGDQYADLFLAAKNLSDAILLSDILRVNTEITKAPLSASMIKRY
DEHHQDLTLLKALVRQQLPEKYKEIFFDQSKNGYAGYIDGGASQEEFYKFIKPILEKMDGTEELL
VKLNREDLLRKQRTFDNGSIPHQIHLGELHAILRRQEDFYPFLKDNREKIEKILTFRIPYYVGPLA
RGNSRFAWMTRKSEETITPWNFEEVVDKGASAQSFIERMTNFDKNLPNEKVLPKHSLLYEYFT
VYNELTKVKYVTEGMRKPAFLSGEQKKAIVDLLFKTNRKVTVKQLKEDYFKKIECFDSVEISGVE

DRFNASLGTYHDLLKIIKDKDFLDNEENEDILEDIVLTLTLFEDREMIEERLKTYAHLFDDKVMKQ
LKRRRYTGWGRLSRKLINGIRDKQSGKTILDFLKSDGFANRNFMQLIHDDSLTFKEDIQKAQVS
GQGDSLHEHIANLAGSPAIKKGILQTVKVVDELVKVMGRHKPENIVIEMARENQTTQKGQKNSR
ERMKRIEEGIKELGSQILKEHPVENTQLQNEKLYLYYLQNGRDMYVDQELDINRLSDYDVDHIV
PQSFLKDDSIDNKVLTRSDKNRGKSDNVPSEEVVKKMKNYWRQLLNAKLITQRKFDNLTKAER
GGLSELDKAGFIKRQLVETRQITKHVAQILDSRMNTKYDENDKLIREVKVITLKSKLVSDFRKDF
QFYKVREINNYHHAHDAYLNAVVGTALIKKYPKLESEFVYGDYKVYDVRKMIAKSEQEIGKATA
KYFFYSNIMNFFKTEITLANGEIRKRPLIETNGETGEIVWDKGRDFATVRKVLSMPQVNIVKKTEV
QTGGFSKESILPKRNSDKLIARKKDWDPKKYGGFDSPTVAYSVLVVAKVEKGKSKKLKSVKELL
GITIMERSSFEKNPIDFLEAKGYKEVKKDLIIKLPKYSLFELENGRKRMLASAGELQKGNELALPS
KYVNFLYLASHYEKLKGSPEDNEQKQLFVEQHKHYLDEIIEQISEFSKRVILADANLDKVLSAYN
KHRDKPIREQAENIIHLFTLTNLGAPAAFKYFDTTIDRKRYTSTKEVLDATLIHQSITGLYETRIDLS
QLGGDSRADSGGSTNLSDIIEKETGKQLVIQESILMLPEEVEEVIGNKPESDILVHTAYDESTDE
NVMLLTSDAPEYKPWALVIQDSNGENKIKMLSGGSPKKKRKV*

ABE-GGGGS$_3$-UGI

MSEVEFSHEYWMRHALTLAKRAWDEREVPVGAVLVHNNRVIGEGWNRPIGRHDPTAHAEIMA
LRQGGLVMQNYRLIDATLYVTLEPCVMCAGAMIHSRIGRVVFGARDAKTGAAGSLMDVLHHPG
MNHRVEITEGILADECAALLSDFFRMRRQEIKAQKKAQSSTDSGGSSGGSSGSETPGTSESAT
PESSGGSSGGSSEVEFSHEYWMRHALTLAKRARDEREVPVGAVLVLNNRVIGEGWNRAIGLH
DPTAHAEIMALRQGGLVMQNYRLIDATLYVTFEPCVMCAGAMIHSRIGRVVFGVRNAKTGAAG
SLMDVLHYPGMNHRVEITEGILADECAALLCYFFRMPRQVFNAQKKAQSSTDASGGGGSGGG
GSGGGGSGTDKKYSIGLAIGTNSVGWAVITDEYKVPSKKFKVLGNTDRHSIKKNLIGALLFDSG
ETAEATRLKRTARRRYTRRKNRICYLQEIFSNEMAKVDDSFFHRLEESFLVEEDKKHERHPIFG
NIVDEVAYHEKYPTIYHLRKKLVDSTDKADLRLIYLALAHMIKFRGHFLIEGDLNPDNSDVDKLFI
QLVQTYNQLFEENPINASGVDAKAILSARLSKSRRLENLIAQLPGEKKNGLFGNLIALSLGLTPNF
KSNFDLAEDAKLQLSKDTYDDDLDNLLAQIGDQYADLFLAAKNLSDAILLSDILRVNTEITKAPLS
ASMIKRYDEHHQDLTLLKALVRQQLPEKYKEIFFDQSKNGYAGYIDGGASQEEFYKFIKPILEKM
DGTEELLVKLNREDLLRKQRTFDNGSIPHQIHLGELHAILRRQEDFYPFLKDNREKIEKILTFRIPY
YVGPLARGNSRFAWMTRKSEETITPWNFEEVVDKGASAQSFIERMTNFDKNLPNEKVLPKHSL
LYEYFTVYNELTKVKYVTEGMRKPAFLSGEQKKAIVDLLFKTNRKVTVKQLKEDYFKKIECFDSV
EISGVEDRFNASLGTYHDLLKIIKDKDFLDNEENEDILEDIVLTLTLFEDREMIEERLKTYAHLFDD
KVMKQLKRRRYTGWGRLSRKLINGIRDKQSGKTILDFLKSDGFANRNFMQLIHDDSLTFKEDIQ
KAQVSGQGDSLHEHIANLAGSPAIKKGILQTVKVVDELVKVMGRHKPENIVIEMARENQTTQKG
QKNSRERMKRIEEGIKELGSQILKEHPVENTQLQNEKLYLYYLQNGRDMYVDQELDINRLSDYD
VDHIVPQSFLKDDSIDNKVLTRSDKNRGKSDNVPSEEVVKKMKNYWRQLLNAKLITQRKFDNLT
KAERGGLSELDKAGFIKRQLVETRQITKHVAQILDSRMNTKYDENDKLIREVKVITLKSKLVSDF
RKDFQFYKVREINNYHHAHDAYLNAVVGTALIKKYPKLESEFVYGDYKVYDVRKMIAKSEQEIG
KATAKYFFYSNIMNFFKTEITLANGEIRKRPLIETNGETGEIVWDKGRDFATVRKVLSMPQVNIVK
KTEVQTGGFSKESILPKRNSDKLIARKKDWDPKKYGGFDSPTVAYSVLVVAKVEKGKSKKLKSV
KELLGITIMERSSFEKNPIDFLEAKGYKEVKKDLIIKLPKYSLFELENGRKRMLASAGELQKGNEL
ALPSKYVNFLYLASHYEKLKGSPEDNEQKQLFVEQHKHYLDEIIEQISEFSKRVILADANLDKVLS
AYNKHRDKPIREQAENIIHLFTLTNLGAPAAFKYFDTTIDRKRYTSTKEVLDATLIHQSITGLYETR
IDLSQLGGDSRADSGGSTNLSDIIEKETGKQLVIQESILMLPEEVEEVIGNKPESDILVHTAYDES
TDENVMLLTSDAPEYKPWALVIQDSNGENKIKMLSGGSPKKKRKV*

ABE-GGGGS$_4$-UGI

MSEVEFSHEYWMRHALTLAKRAWDEREVPVGAVLVHNNRVIGEGWNRPIGRHDPTAHAEIMA
LRQGGLVMQNYRLIDATLYVTLEPCVMCAGAMIHSRIGRVVFGARDAKTGAAGSLMDVLHHPG
MNHRVEITEGILADECAALLSDFFRMRRQEIKAQKKAQSSTDSGGSSGGSSGSETPGTSESAT
PESSGGSSGGSSEVEFSHEYWMRHALTLAKRARDEREVPVGAVLVLNNRVIGEGWNRAIGLH
DPTAHAEIMALRQGGLVMQNYRLIDATLYVTFEPCVMCAGAMIHSRIGRVVFGVRNAKTGAAG
SLMDVLHYPGMNHRVEITEGILADECAALLCYFFRMPRQVFNAQKKAQSSTDASGGGGSGGG
GSGGGGSGGGGSGTDKKYSIGLAIGTNSVGWAVITDEYKVPSKKFKVLGNTDRHSIKKNLIGAL
LFDSGETAEATRLKRTARRRYTRRKNRICYLQEIFSNEMAKVDDSFFHRLEESFLVEEDKKHER
HPIFGNIVDEVAYHEKYPTIYHLRKKLVDSTDKADLRLIYLALAHMIKFRGHFLIEGDLNPDNSDV
DKLFIQLVQTYNQLFEENPINASGVDAKAILSARLSKSRRLENLIAQLPGEKKNGLFGNLIALSLG
LTPNFKSNFDLAEDAKLQLSKDTYDDDLDNLLAQIGDQYADLFLAAKNLSDAILLSDILRVNTEIT
KAPLSASMIKRYDEHHQDLTLLKALVRQQLPEKYKEIFFDQSKNGYAGYIDGGASQEEFYKFIKP
ILEKMDGTEELLVKLNREDLLRKQRTFDNGSIPHQIHLGELHAILRRQEDFYPFLKDNREKIEKIL
TFRIPYYVGPLARGNSRFAWMTRKSEETITPWNFEEVVDKGASAQSFIERMTNFDKNLPNEKV
LPKHSLLYEYFTVYNELTKVKYVTEGMRKPAFLSGEQKKAIVDLLFKTNRKVTVKQLKEDYFKKI
ECFDSVEISGVEDRFNASLGTYHDLLKIIKDKDFLDNEENEDILEDIVLTLTLFEDREMIEERLKTY
AHLFDDKVMKQLKRRRYTGWGRLSRKLINGIRDKQSGKTILDFLKSDGFANRNFMQLIHDDSLT
FKEDIQKAQVSGQGDSLHEHIANLAGSPAIKKGILQTVKVVDELVKVMGRHKPENIVIEMARENQ
TTQKGQKNSRERMKRIEEGIKELGSQILKEHPVENTQLQNEKLYLYYLQNGRDMYVDQELDINR
LSDYDVDHIVPQSFLKDDSIDNKVLTRSDKNRGKSDNVPSEEVVKKMKNYWRQLLNAKLITQR
KFDNLTKAERGGLSELDKAGFIKRQLVETRQITKHVAQILDSRMNTKYDENDKLIREVKVITLKSK
LVSDFRKDFQFYKVREINNYHHAHDAYLNAVVGTALIKKYPKLESEFVYGDYKVYDVRKMIAKS
EQEIGKATAKYFFYSNIMNFFKTEITLANGEIRKRPLIETNGETGEIVWDKGRDFATVRKVLSMP
QVNIVKKTEVQTGGFSKESILPKRNSDKLIARKKDWDPKKYGGFDSPTVAYSVLVVAKVEKGKS
KKLKSVKELLGITIMERSSFEKNPIDFLEAKGYKEVKKDLIIKLPKYSLFELENGRKRMLASAGEL
QKGNELALPSKYVNFLYLASHYEKLKGSPEDNEQKQLFVEQHKHYLDEIIEQISEFSKRVILADA
NLDKVLSAYNKHRDKPIREQAENIIHLFTLTNLGAPAAFKYFDTTIDRKRYTSTKEVLDATLIHQSI
TGLYETRIDLSQLGGDSRADSGGSTNLSDIIEKETGKQLVIQESILMLPEEVEEVIGNKPESDILV
HTAYDESTDENVMLLTSDAPEYKPWALVIQDSNGENKIKMLSGGSPKKKRKV*

ABE-GGGGS$_5$-UGI

MSEVEFSHEYWMRHALTLAKRAWDEREVPVGAVLVHNNRVIGEGWNRPIGRHDPTAHAEIMA
LRQGGLVMQNYRLIDATLYVTLEPCVMCAGAMIHSRIGRVVFGARDAKTGAAGSLMDVLHHPG
MNHRVEITEGILADECAALLSDFFRMRRQEIKAQKKAQSSTDSGGSSGGSSGSETPGTSESAT
PESSGGSSGGSSEVEFSHEYWMRHALTLAKRARDEREVPVGAVLVLNNRVIGEGWNRAIGLH
DPTAHAEIMALRQGGLVMQNYRLIDATLYVTFEPCVMCAGAMIHSRIGRVVFGVRNAKTGAAG
SLMDVLHYPGMNHRVEITEGILADECAALLCYFFRMPRQVFNAQKKAQSSTDASGGGGSGGG
GSGGGGSGGGGSGGGGSGTDKKYSIGLAIGTNSVGWAVITDEYKVPSKKFKVLGNTDRHSIK
KNLIGALLFDSGETAEATRLKRTARRRYTRRKNRICYLQEIFSNEMAKVDDSFFHRLEESFLVEE
DKKHERHPIFGNIVDEVAYHEKYPTIYHLRKKLVDSTDKADLRLIYLALAHMIKFRGHFLIEGDLN
PDNSDVDKLFIQLVQTYNQLFEENPINASGVDAKAILSARLSKSRRLENLIAQLPGEKKNGLFGN
LIALSLGLTPNFKSNFDLAEDAKLQLSKDTYDDDLDNLLAQIGDQYADLFLAAKNLSDAILLSDILR
VNTEITKAPLSASMIKRYDEHHQDLTLLKALVRQQLPEKYKEIFFDQSKNGYAGYIDGGASQEEF
YKFIKPILEKMDGTEELLVKLNREDLLRKQRTFDNGSIPHQIHLGELHAILRRQEDFYPFLKDNRE
KIEKILTFRIPYYVGPLARGNSRFAWMTRKSEETITPWNFEEVVDKGASAQSFIERMTNFDKNLP
NEKVLPKHSLLYEYFTVYNELTKVKYVTEGMRKPAFLSGEQKKAIVDLLFKTNRKVTVKQLKED
YFKKIECFDSVEISGVEDRFNASLGTYHDLLKIIKDKDFLDNEENEDILEDIVLTLTLFEDREMIEE
RLKTYAHLFDDKVMKQLKRRRYTGWGRLSRKLINGIRDKQSGKTILDFLKSDGFANRNFMQLIH
DDSLTFKEDIQKAQVSGQGDSLHEHIANLAGSPAIKKGILQTVKVVDELVKVMGRHKPENIVIEM

ARENQTTQKGQKNSRERMKRIEEGIKELGSQILKEHPVENTQLQNEKLYLYYLQNGRDMYVDQ
ELDINRLSDYDVDHIVPQSFLKDDSIDNKVLTRSDKNRGKSDNVPSEEVVKKMKNYWRQLLNA
KLITQRKFDNLTKAERGGLSELDKAGFIKRQLVETRQITKHVAQILDSRMNTKYDENDKLIREVK
VITLKSKLVSDFRKDFQFYKVREINNYHHAHDAYLNAVVGTALIKKYPKLESEFVYGDYKVYDVR
KMIAKSEQEIGKATAKYFFYSNIMNFFKTEITLANGEIRKRPLIETNGETGEIVWDKGRDFATVRK
VLSMPQVNIVKKTEVQTGGFSKESILPKRNSDKLIARKKDWDPKKYGGFDSPTVAYSVLVVAKV
EKGKSKKLKSVKELLGITIMERSSFEKNPIDFLEAKGYKEVKKDLIIKLPKYSLFELENGRKRMLA
SAGELQKGNELALPSKYVNFLYLASHYEKLKGSPEDNEQKQLFVEQHKHYLDEIIEQISEFSKRV
ILADANLDKVLSAYNKHRDKPIREQAENIIHLFTLTNLGAPAAFKYFDTTIDRKRYTSTKEVLDATL
IHQSITGLYETRIDLSQLGGDSRADSGGSTNLSDIIEKETGKQLVIQESILMLPEEVEEVIGNKPES
DILVHTAYDESTDENVMLLTSDAPEYKPWALVIQDSNGENKIKMLSGGSPKKKRKV*

ABE-$GGGGS_6$-UGI

MSEVEFSHEYWMRHALTLAKRAWDEREVPVGAVLVHNNRVIGEGWNRPIGRHDPTAHAEIMA
LRQGGLVMQNYRLIDATLYVTLEPCVMCAGAMIHSRIGRVVFGARDAKTGAAGSLMDVLHHPG
MNHRVEITEGILADECAALLSDFFRMRRQEIKAQKKAQSSTDSGGSSGGSSGSETPGTSESAT
PESSGGSSGGSSEVEFSHEYWMRHALTLAKRARDEREVPVGAVLVLNNRVIGEGWNRAIGLH
DPTAHAEIMALRQGGLVMQNYRLIDATLYVTFEPCVMCAGAMIHSRIGRVVFGVRNAKTGAAG
SLMDVLHYPGMNHRVEITEGILADECAALLCYFFRMPRQVFNAQKKAQSSTDASGGGGSGGG
GSGGGGSGGGGSGGGGSGGGGSGTDKKYSIGLAIGTNSVGWAVITDEYKVPSKKFKVLGNT
DRHSIKKNLIGALLFDSGETAEATRLKRTARRRYTRRKNRICYLQEIFSNEMAKVDDSFFHRLEE
SFLVEEDKKHERHPIFGNIVDEVAYHEKYPTIYHLRKKLVDSTDKADLRLIYLALAHMIKFRGHFLI
EGDLNPDNSDVDKLFIQLVQTYNQLFEENPINASGVDAKAILSARLSKSRRLENLIAQLPGEKKN
GLFGNLIALSLGLTPNFKSNFDLAEDAKLQLSKDTYDDDLDNLLAQIGDQYADLFLAAKNLSDAIL
LSDILRVNTEITKAPLSASMIKRYDEHHQDLTLLKALVRQQLPEKYKEIFFDQSKNGYAGYIDGG
ASQEEFYKFIKPILEKMDGTEELLVKLNREDLLRKQRTFDNGSIPHQIHLGELHAILRRQEDFYPF
LKDNREKIEKILTFRIPYYVGPLARGNSRFAWMTRKSEETITPWNFEEVVDKGASAQSFIERMT
NFDKNLPNEKVLPKHSLLYEYFTVYNELTKVKYVTEGMRKPAFLSGEQKKAIVDLLFKTNRKVT
VKQLKEDYFKKIECFDSVEISGVEDRFNASLGTYHDLLKIIKDKDFLDNEENEDILEDIVLTLTLFE
DREMIEERLKTYAHLFDDKVMKQLKRRRYTGWGRLSRKLINGIRDKQSGKTILDFLKSDGFANR
NFMQLIHDDSLTFKEDIQKAQVSGQGDSLHEHIANLAGSPAIKKGILQTVKVVDELVKVMGRHK
PENIVIEMARENQTTQKGQKNSRERMKRIEEGIKELGSQILKEHPVENTQLQNEKLYLYYLQNG
RDMYVDQELDINRLSDYDVDHIVPQSFLKDDSIDNKVLTRSDKNRGKSDNVPSEEVVKKMKNY
WRQLLNAKLITQRKFDNLTKAERGGLSELDKAGFIKRQLVETRQITKHVAQILDSRMNTKYDEN
DKLIREVKVITLKSKLVSDFRKDFQFYKVREINNYHHAHDAYLNAVVGTALIKKYPKLESEFVYG
DYKVYDVRKMIAKSEQEIGKATAKYFFYSNIMNFFKTEITLANGEIRKRPLIETNGETGEIVWDKG
RDFATVRKVLSMPQVNIVKKTEVQTGGFSKESILPKRNSDKLIARKKDWDPKKYGGFDSPTVAY
SVLVVAKVEKGKSKKLKSVKELLGITIMERSSFEKNPIDFLEAKGYKEVKKDLIIKLPKYSLFELEN
GRKRMLASAGELQKGNELALPSKYVNFLYLASHYEKLKGSPEDNEQKQLFVEQHKHYLDEIIEQ
ISEFSKRVILADANLDKVLSAYNKHRDKPIREQAENIIHLFTLTNLGAPAAFKYFDTTIDRKRYTST
KEVLDATLIHQSITGLYETRIDLSQLGGDSRADSGGSTNLSDIIEKETGKQLVIQESILMLPEEVEE
VIGNKPESDILVHTAYDESTDENVMLLTSDAPEYKPWALVIQDSNGENKIKMLSGGSPKKKRKV
*

ABE-$GGGGS_7$-UGI

MSEVEFSHEYWMRHALTLAKRAWDEREVPVGAVLVHNNRVIGEGWNRPIGRHDPTAHAEIMA
LRQGGLVMQNYRLIDATLYVTLEPCVMCAGAMIHSRIGRVVFGARDAKTGAAGSLMDVLHHPG
MNHRVEITEGILADECAALLSDFFRMRRQEIKAQKKAQSSTDSGGSSGGSSGSETPGTSESAT

PESSGGSSGGSSEVEFSHEYWMRHALTLAKRARDEREVPVGAVLVLNNRVIGEGWNRAIGLH
DPTAHAEIMALRQGGLVMQNYRLIDATLYVTFEPCVMCAGAMIHSRIGRVVFGVRNAKTGAAG
SLMDVLHYPGMNHRVEITEGILADECAALLCYFFRMPRQVFNAQKKAQSSTDASGGGGSGGG
GSGGGGSGGGGSGGGGSGGGGSGGGGSGTDKKYSIGLAIGTNSVGWAVITDEYKVPSKKFK
VLGNTDRHSIKKNLIGALLFDSGETAEATRLKRTARRRYTRRKNRICYLQEIFSNEMAKVDDSFF
HRLEESFLVEEDKKHERHPIFGNIVDEVAYHEKYPTIYHLRKKLVDSTDKADLRLIYLALAHMIKF
RGHFLIEGDLNPDNSDVDKLFIQLVQTYNQLFEENPINASGVDAKAILSARLSKSRRLENLIAQLP
GEKKNGLFGNLIALSLGLTPNFKSNFDLAEDAKLQLSKDTYDDDLDNLLAQIGDQYADLFLAAKN
LSDAILLSDILRVNTEITKAPLSASMIKRYDEHHQDLTLLKALVRQQLPEKYKEIFFDQSKNGYAG
YIDGGASQEEFYKFIKPILEKMDGTEELLVKLNREDLLRKQRTFDNGSIPHQIHLGELHAILRRQE
DFYPFLKDNREKIEKILTFRIPYYVGPLARGNSRFAWMTRKSEETITPWNFEEVVDKGASAQSFI
ERMTNFDKNLPNEKVLPKHSLLYEYFTVYNELTKVKYVTEGMRKPAFLSGEQKKAIVDLLFKTN
RKVTVKQLKEDYFKKIECFDSVEISGVEDRFNASLGTYHDLLKIIKDKDFLDNEENEDILEDIVLTL
TLFEDREMIEERLKTYAHLFDDKVMKQLKRRRYTGWGRLSRKLINGIRDKQSGKTILDFLKSDG
FANRNFMQLIHDDSLTFKEDIQKAQVSGQGDSLHEHIANLAGSPAIKKGILQTVKVVDELVKVM
GRHKPENIVIEMARENQTTQKGQKNSRERMKRIEEGIKELGSQILKEHPVENTQLQNEKLYLYY
LQNGRDMYVDQELDINRLSDYDVDHIVPQSFLKDDSIDNKVLTRSDKNRGKSDNVPSEEVVKK
MKNYWRQLLNAKLITQRKFDNLTKAERGGLSELDKAGFIKRQLVETRQITKHVAQILDSRMNTK
YDENDKLIREVKVITLKSKLVSDFRKDFQFYKVREINNYHHAHDAYLNAVVGTALIKKYPKLESEF
VYGDYKVYDVRKMIAKSEQEIGKATAKYFFYSNIMNFFKTEITLANGEIRKRPLIETNGETGEIVW
DKGRDFATVRKVLSMPQVNIVKKTEVQTGGFSKESILPKRNSDKLIARKKDWDPKKYGGFDSP
TVAYSVLVVAKVEKGKSKKLKSVKELLGITIMERSSFEKNPIDFLEAKGYKEVKKDLIIKLPKYSLF
ELENGRKRMLASAGELQKGNELALPSKYVNFLYLASHYEKLKGSPEDNEQKQLFVEQHKHYLD
EIIEQISEFSKRVILADANLDKVLSAYNKHRDKPIREQAENIIHLFTLTNLGAPAAFKYFDTTIDRKR
YTSTKEVLDATLIHQSITGLYETRIDLSQLGGDSRADSGGSTNLSDIIEKETGKQLVIQESILMLPE
EVEEVIGNKPESDILVHTAYDESTDENVMLLTSDAPEYKPWALVIQDSNGENKIKMLSGGSPKK
KRKV*

N-ABE

MSEVEFSHEYWMRHALTLAKRAWDEREVPVGAVLVHNNRVIGEGWNRPIGRHDPTAHAEIMA
LRQGGLVMQNYRLIDATLYVTLEPCVMCAGAMIHSRIGRVVFGARDAKTGAAGSLMDVLHHPG
MNHRVEITEGILADECAALLSDFFRMRRQEIKAQKKAQSSTDSGGSSGGSSGSETPGTSESAT
PESSGGSSGGSSEVEFSHEYWMRHALTLAKRARDEREVPVGAVLVLNNRVIGEGWNRAIGLH
DPTAHAEIMALRQGGLVMQNYRLIDATLYVTFEPCVMCAGAMIHSRIGRVVFGVRNAKTGAAG
SLMDVLHYPGMNHRVEITEGILADECAALLCYFFRMPRQVFNAQKKAQSSTDSGGSSGGSSG
SETPGTSESATPESSGGSSGGSDKKYSIGLAIGTNSVGWAVITDEYKVPSKKFKVLGNTDRHSI
KKNLIGALLFDSGETAEATRLKRTARRRYTRRKNRICYLQEIFSNEMAKVDDSFFHRLEESFLVE
EDKKHERHPIFGNIVDEVAYHEKYPTIYHLRKKLVDSTDKADLRLIYLALAHMIKFRGHFLIEGDL
NPDNSDVDKLFIQLVQTYNQLFEENPINASGVDAKAILSARLSKSRRLENLIAQLPGEKKNGLFG
NLIALSLGLTPNFKSNFDLAEDAKLQLSKDTYDDDLDNLLAQIGDQYADLFLAAKNLSDAILLSDIL
RVNTEITKAPLSASMIKRYDEHHQDLTLLKALVRQQLPEKYKEIFFDQSKNGYAGYIDGGASQE
EFYKFIKPILEKMDGTEELLVKLNREDLLRKQRTFDNGSIPHQIHLGELHAILRRQEDFYPFLKDN
REKIEKILTFRIPYYVGPLARGNSRFAWMTRKSEETITPWNFEEVVDKGASAQSFIERMTNFDK
NLPNEKVLPKHSLLYEYFTVYNELTKVKYVTEGMRKPAFLSGEQKKAIVDLLFKTNRKVTVKQL
KEDYFKKIECFDSVEISGVEDRFNASLGTYHDLLKIIKDKDFLDNEENEDILEDIVLTLTLFEDREM
IEERLKTYAHLFDDKVMKQLKRRRYTGWGRLSRKLINGIRDKQSGKTILDFLKSDGFANRNFMQ
LIHDDSLTFKEDIQKAQVCLAGDTLITLADGRRVPIRELVSQQNFSVWALNPQTYRLERARVSRA
FCTGIKPVYRLTTRLGRSIRATANHRFLTPQGWKRVDELQPGDYLALPRRIPTAS*

C-ABE

MAAACPELRQLAQSDVYWDPIVSIEPDGVEEVFDLTVPGPHNFVANDIIAHNSGQGDSLHEHIA
NLAGSPAIKKGILQTVKVVDELVKVMGRHKPENIVIEMARENQTTQKGQKNSRERMKRIEEGIK
ELGSQILKEHPVENTQLQNEKLYLYYLQNGRDMYVDQELDINRLSDYDVDHIVPQSFLKDDSID
NKVLTRSDKNRGKSDNVPSEEVVKKMKNYWRQLLNAKLITQRKFDNLTKAERGGLSELDKAGF
IKRQLVETRQITKHVAQILDSRMNTKYDENDKLIREVKVITLKSKLVSDFRKDFQFYKVREINNYH
HAHDAYLNAVVGTALIKKYPKLESEFVYGDYKVYDVRKMIAKSEQEIGKATAKYFFYSNIMNFFK
TEITLANGEIRKRPLIETNGETGEIVWDKGRDFATVRKVLSMPQVNIVKKTEVQTGGFSKESILP
KRNSDKLIARKKDWDPKKYGGFDSPTVAYSVLVVAKVEKGKSKKLKSVKELLGITIMERSSFEK
NPIDFLEAKGYKEVKKDLIIKLPKYSLFELENGRKRMLASAGELQKGNELALPSKYVNFLYLASH
YEKLKGSPEDNEQKQLFVEQHKHYLDEIIEQISEFSKRVILADANLDKVLSAYNKHRDKPIREQA
ENIIHLFTLTNLGAPAAFKYFDTTIDRKRYTSTKEVLDATLIHQSITGLYETRIDLSQLGGDSRADP
KKKRKV*

N-ABE-AAV

MSEVEFSHEYWMRHALTLAKRAWDEREVPVGAVLVHNNRVIGEGWNRPIGRHDPTAHAEIMA
LRQGGLVMQNYRLIDATLYVTLEPCVMCAGAMIHSRIGRVVFGARDAKTGAAGSLMDVLHHPG
MNHRVEITEGILADECAALLSDFFRMRRQEIKAQKKAQSSTDSGGSSGGSSGSETPGTSESAT
PESSGGSSGGSSEVEFSHEYWMRHALTLAKRARDEREVPVGAVLVLNNRVIGEGWNRAIGLH
DPTAHAEIMALRQGGLVMQNYRLIDATLYVTFEPCVMCAGAMIHSRIGRVVFGVRNAKTGAAG
SLMDVLHYPGMNHRVEITEGILADECAALLCYFFRMPRQVFNAQKKAQSSTDASGGGGSGGG
GSGGGGSGGGGSGGGGSGTDKKYSIGLAIGTNSVGWAVITDEYKVPSKKFKVLGNTDRHSIK
KNLIGALLFDSGETAEATRLKRTARRRYTRRKNRICYLQEIFSNEMAKVDDSFFHRLEESFLVEE
DKKHERHPIFGNIVDEVAYHEKYPTIYHLRKKLVDSTDKADLRLIYLALAHMIKFRGHFLIEGDLN
PDNSDVDKLFIQLVQTYNQLFEENPINASGVDAKAILSARLSKSRRLENLIAQLPGEKKNGLFGN
LIALSLGLTPNFKSNFDLAEDAKLQLSKDTYDDDLDNLLAQIGDQYADLFLAAKNLSDAILLSDILR
VNTEITKAPLSASMIKRYDEHHQDLTLLKALVRQQLPEKYKEIFFDQSKNGYAGYIDGGASQEEF
YKFIKPILEKMDGTEELLVKLNREDLLRKQRTFDNGSIPHQIHLGELHAILRRQEDFYPFLKDNRE
KIEKILTFRIPYYVGPLARGNSRFAWMTRKSEETITPWNFEEVVDKGASAQSFIERMTNFDKNLP
NEKVLPKHSLLYEYFTVYNELTKVKYVTEGMRKPAFLSGEQKKAIVDLLFKTNRKVTVKQLKED
YFKKIECFDSVEISGVEDRFNASLGTYHDLLKIIKDKDFLDNEENEDILEDIVLTLTLFEDREMIEE
RLKTYAHLFDDKVMKQLKRRRYTGWGRLSRKLINGIRDKQSGKTILDFLKSDGFANRNFMQLIH
DDSLTFKEDIQKAQVCLAGDTLITLADGRRVPIRELVSQQNFSVWALNPQTYRLERARVSRAFC
TGIKPVYRLTTRLGRSIRATANHRFLTPQGWKRVDELQPGDYLALPRRIPTAS*

C-ABE-AAV

MAAACPELRQLAQSDVYWDPIVSIEPDGVEEVFDLTVPGPHNFVANDIIAHNSGQGDSLHEHIA
NLAGSPAIKKGILQTVKVVDELVKVMGRHKPENIVIEMARENQTTQKGQKNSRERMKRIEEGIK
ELGSQILKEHPVENTQLQNEKLYLYYLQNGRDMYVDQELDINRLSDYDVDHIVPQSFLKDDSID
NKVLTRSDKNRGKSDNVPSEEVVKKMKNYWRQLLNAKLITQRKFDNLTKAERGGLSELDKAGF
IKRQLVETRQITKHVAQILDSRMNTKYDENDKLIREVKVITLKSKLVSDFRKDFQFYKVREINNYH
HAHDAYLNAVVGTALIKKYPKLESEFVYGDYKVYDVRKMIAKSEQEIGKATAKYFFYSNIMNFFK
TEITLANGEIRKRPLIETNGETGEIVWDKGRDFATVRKVLSMPQVNIVKKTEVQTGGFSKESILP
KRNSDKLIARKKDWDPKKYGGFDSPTVAYSVLVVAKVEKGKSKKLKSVKELLGITIMERSSFEK
NPIDFLEAKGYKEVKKDLIIKLPKYSLFELENGRKRMLASAGELQKGNELALPSKYVNFLYLASH
YEKLKGSPEDNEQKQLFVEQHKHYLDEIIEQISEFSKRVILADANLDKVLSAYNKHRDKPIREQA
ENIIHLFTLTNLGAPAAFKYFDTTIDRKRYTSTKEVLDATLIHQSITGLYETRIDLSQLGGDSGGST

NLSDIIEKETGKQLVIQESILMLPEEVEEVIGNKPESDILVHTAYDESTDENVMLLTSDAPEYKPW
ALVIQDSNGENKIKMLSYPYDVPDYAYPYDVPDYAYPYDVPDYASGGSPKKKRKV*

References

1 Hsu, P. D. *et al.* DNA targeting specificity of RNA-guided Cas9 nucleases. *Nat Biotechnol* **31**, 827-832, doi:10.1038/nbt.2647 (2013).

2 Doench, J. G. *et al.* Optimized sgRNA design to maximize activity and minimize off-target effects of CRISPR-Cas9. *Nat Biotechnol* **34**, 184-191, doi:10.1038/nbt.3437 (2016).
